# Pulse consumption improves indices of glycemic control in adults with and without type 2 diabetes: a systematic review and meta-analysis of acute and long-term randomized controlled trials

**DOI:** 10.1007/s00394-021-02685-y

**Published:** 2021-09-29

**Authors:** Maryam S. Hafiz, Matthew D. Campbell, Lauren L. O’Mahoney, Melvin Holmes, Caroline Orfila, Christine Boesch

**Affiliations:** 1 School of Food Science and Nutrition, University of Leeds, Leeds, LS2 9JT UK; 2 Faculty of Applied Medical Sciences, Department of Clinical Nutrition, King Abdul-Aziz University, Jeddah, Saudi Arabia; 3 School of Nursing and Health Sciences, Faculty of Health Sciences and Wellbeing, University of Sunderland, Sunderland, UK; 4 Wellcome-MRC Institute of Metabolic Science, University of Cambridge, Cambridge, UK; 5 Leeds Institute of Cardiovascular and Metabolic Medicine, University of Leeds, Leeds, UK; 6 Leicester Diabetes Centre, University of Leicester, Leicester, UK

**Keywords:** Pulses, Glucose, Diabetes, Postprandial glycemia, Systematic review, Meta-analysis

## Abstract

**Purpose:**

Findings from randomized controlled trials (RCTs) evaluating the effect of pulse intake on glycemic control are inconsistent and conclusive evidence is lacking. The aim of this study was to systematically review the impact of pulse consumption on post-prandial and long-term glycemic control in adults with and without type 2 diabetes (T2D).

**Methods:**

Databases were searched for RCTs, reporting outcomes of post-prandial and long-term interventions with different pulse types on parameters of glycemic control in normoglycemic and T2D adults. Effect size (ES) was calculated using random effect model and meta-regression was conducted to assess the impact of various moderator variables such as pulse type, form, dose, and study duration on ES.

**Results:**

From 3334 RCTs identified, 65 studies were eligible for inclusion involving 2102 individuals. In acute RCTs, pulse intake significantly reduced peak post-prandial glucose concentration in participants with T2D (ES  – 2.90; 95%CI  – 4.60,  – 1.21; *p* ≤ 0.001; *I*^2^ = 93%) and without T2D (ES  – 1.38; 95%CI  – 1.78,  – 0.99; *p* ≤ 0.001; *I*^2^ = 86%). Incorporating pulse consumption into long-term eating patterns significantly attenuated fasting glucose in normoglycemic adults (ES  – 0.06; 95%CI  – 0.12, 0.00; *p* ≤ 0.05; *I*^2^ = 30%). Whereas, in T2D participants, pulse intake significantly lowered fasting glucose (ES  – 0.54; 95%CI  – 0.83,  – 0.24; *p* ≤ 0.001; *I*^2^ = 78%), glycated hemoglobin A1c (HbA_1c_) (ES  – 0.17; 95%CI  – 0.33, 0.00; *p* ≤ 0.05; *I*^2^ = 78) and homeostatic model assessment of insulin resistance (HOMA-IR) (ES  – 0.47; 95%CI  – 1.25,  – 0.31; *p* ≤ 0.05; *I*^2^ = 79%).

**Conclusion:**

Pulse consumption significantly reduced acute post-prandial glucose concentration > 1 mmol/L in normoglycemic adults and > 2.5 mmol/L in those with T2D, and improved a range of long-term glycemic control parameters in adults with and without T2D.

**PROSPERO registry number:**

(CRD42019162322).

**Supplementary Information:**

The online version contains supplementary material available at 10.1007/s00394-021-02685-y.

## Introduction

The European Association for the Study of Diabetes (EASD) and the American Diabetes Association (ADA) advocate increasing fiber intake, specifically through the consumption of pulses as a means to improve blood glucose control in adults with and without T2D [1, 2]. Several epidemiological studies have reported inverse associations between pulse intake and incidence of T2D [3, 4]. In addition, RCTs suggest that pulse consumption may improve acute post-prandial glucose control, and lower fasting blood glucose, insulin and HbA_1c_ levels when incorporated into long-term eating patterns [5, 6].

Pulses are rich sources of low glycemic index (GI) carbohydrates (CHO, up to 65%), and protein with up to 25% (dry weight) [7]. Low GI, fiber-rich foods have been shown to reduce post-prandial glycemic responses (PPGR) compared to foods with similar CHO content [8, 9], as well as protein addition to breakfast is suggested to improve PPGR [10]. In addition, pulses contain phytochemicals such as catechins and procyanidins which have been demonstrated to suppress the enzymatic activity of CHO digestive enzymes including α-amylase and α-glucosidase thereby contributing towards improved post-prandial glycemic control [11–13].

A number of randomized controlled trials have assessed the effect of pulse intake on acute post-prandial and long-term glucose response [14–23]. The studies differed in the type of pulses used, processing, doses and control group, and in different volunteer profiles [6, 24–33]. The study outcomes vary considerably with low quality of evidence and, therefore, the true effect size of pulse intake on measures of glycemic handling remains unclear [34]. A previous systematic review and meta-analysis by Sievenpiper et al. (2009) concluded a significant reduction in fasting blood glucose and insulin after long-term consumption of pulses alone, as part of low GI or high-fiber diets [35]. However, the review was published in 2009 and only long-term trials were included in their review. Considering that there are more than 20 long-term trials published since 2009 and given the lack of summarized evidence on post-prandial glucose response after intake of pulses, the aim of the current systematic review is to update the evidence on long-term effects of pulse consumption on glycemic indices as well as integrate the acute glucose response along from RCTs on individuals with and without T2D.

## Methods

The guidelines of Preferred Reporting Items for Systematic Reviews and Meta-Analyses (PRISMA) [36] were followed for conducting this systematic review and meta-analysis. The systematic review was prospectively registered with PROSPERO (CRD42019162322).

### Search strategy and study selection

We searched Pubmed and Cochrane library databases to identify all randomized clinical trials (RCTs) conducted and relevant to the topic until 28th of January 2021. Full search terms are illustrated in Supplemental Table 1. No filters for language, date of publication, or design of the study were applied when searching the databases. An additional manual search was conducted through reviewing reference lists of selected articles and reviews.

The study selection process was performed in duplicate independently by two reviewers by initially reviewing the titles and abstracts and finally reviewing the full texts to identify all eligible RCTs. Included studies were randomized controlled trials either acute (assessing single meal response) or long-term (assessing intake > 2 weeks) [37], including all adults except type 1 diabetes mellitus and gestational diabetes, investigating the effect of intake of pulses in comparison to control diet, on parameters of glycemic control measured using capillary or venous blood. Studies were excluded if they investigated legumes other than pulses such as soya beans or green peas, failed to use a matched available carbohydrate control in acute glucose response trials; the pattern of pulse consumption was not specified; used pulse fractions such as their extracts; protein isolates or husk only; reported subsequent second meal effect rather than immediate response; did not exclude or account for confounding factors whether in participants or intervention diets that might impact glucose metabolism; or outcome measures of glycemic control were not reported. In studies where different interventions were used in different arms, only data from arms that met the eligibility criteria were included in the analysis. Included trials were limited to published and peer-reviewed RCTs available as full texts in English. Corresponding authors were contacted to request the full text in cases where the full text was not available online before deciding on exclusion.

### Data extraction and quality assessment

Data were extracted by single author and included: first author and year of publication; publishing journal; design of the study; intervention arms; number of visits in acute studies; study duration in long-term studies; sample size and participant characteristics (gender, health status, age group and body mass index); intervention design and control (type, dose and format); pulse characteristics (type, dose and physical form). The outcome measures of acute trials were extracted for means and standard deviations of baseline and post-prandial glucose (mmol/L) and insulin (mIU/L) values and their area under the curves (AUCs). In the long-term trials, baseline and post-intervention mean and standard deviation values were extracted for fasting blood glucose (mmol/L), insulin (mIU/L), glycated hemoglobin (%) and insulin resistance expressed as HOMA-IR. Where data were presented in non-standard units, they were converted to standard reporting units. If data were available in figure format only, values were digitized using Graph Digitizer. In trials not reporting the standard deviation, the values were derived from standard errors or confidence intervals (CI).

Bias assessment of individual trials was performed independently by two reviewers following the updated Cochrane Collaboration’s tool for assessing risk of bias (RoB2) [38]. The trials were classified into three categories “high risk, low risk, or some concerns raised” in five domains which are as follows: randomization process, deviations from intended interventions, missing outcome data, measurement of the outcome, and selection of the reported results. The proposed algorithm was followed in signaling questions to judge risk of bias of each domain as well as overall risk of bias. Publication bias was visually assessed by inspection of funnel plots and quantitatively using Egger’s test for each outcome [39].

### Data analysis

Data were analyzed using Review Manager (RevMan) 5.3.5 Copenhagen: The Nordic Cochrane Centre, The Cochrane Collaboration, 2014; and R Core Team (2020), R: A language and environment for statistical computing, R foundation for statistical computing, Vienna, Austria. The random effects model was chosen assuming that the RCTs included in the analysis were functionally inequivalent. Weighted averages were calculated in trials using more than one arm for intervention to avoid errors in analyses [40]. RCTs not reporting the amount of pulses administered were excluded from the meta-analysis. Pooled random effects analyses were performed to estimate the effect size in acute and long-term RCTs on normoglycemic and T2D participants. The entered data included sample size, reported means and standard deviations for intervention arms and their matched carbohydrate controls of each trial. Effect size was estimated for post-prandial glucose and insulin response in acute RCTs and for the difference between pre- and post-intervention in fasting blood glucose, insulin, glycated hemoglobin, and HOMA-IR values as raw mean differences and 95% CIs. A negative ES was interpreted as favoring pulse intake, while a positive ES favored control. The inter-study variance was assessed using tau^2^ and I^2^ along with calculation of prediction intervals (PI). Sensitivity analysis was performed to explore the impact of removing one RCT on outcomes, as well as investigate removal of studies with high risk of bias on ES [41].

Subgroup analysis and meta-regression were performed if ≥ 10 RCTs could be included in the meta-analysis to explore the variations in ES, considering pulse type or processing method used in intervention arms, control food used for comparison, and dose or duration of the study as variables [41].

### Grading the evidence

The Grading of Recommendations Assessment, Development and Evaluation (GRADE) tool was conducted by single author for interpreting outcome data to evaluate the certainty of evidence [42]. Evidences on the ES can be graded to ‘very low’, ‘low’, ‘moderate’, or ‘high’ based on evaluation outcomes in five domains. The domains are as follows: overall risk of bias, inconsistency, indirectness, imprecision, and other considerations.

## Results

**Fig. 1:**
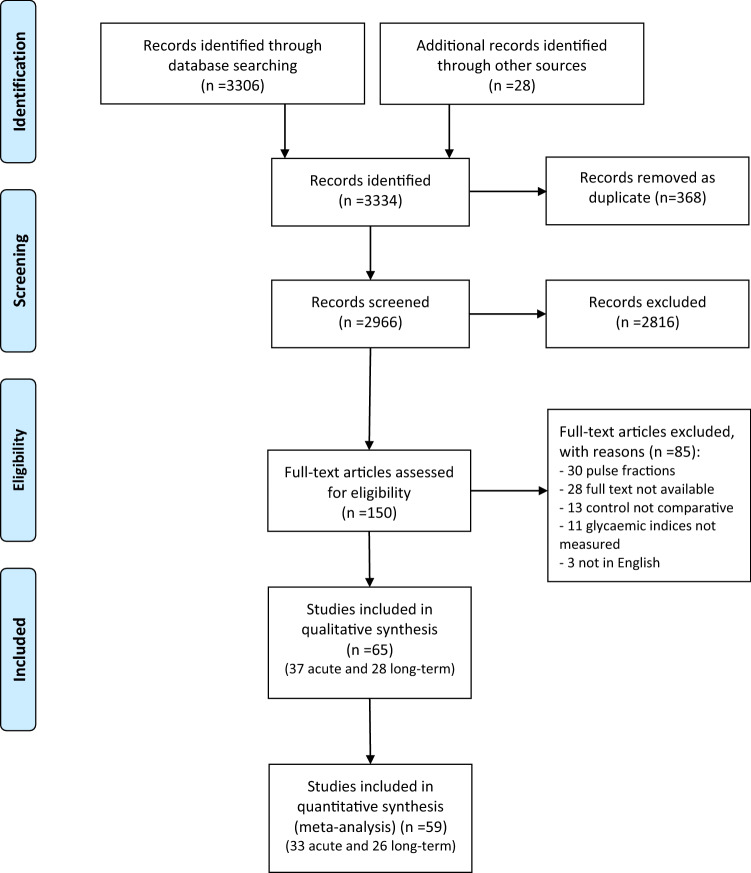
Flow diagram of trial selection

A total of 3334 studies were identified through database searches and additional sources, of which 2966 were screened based on title and abstract only. Of these, 150 studies were reviewed as full text and subsequently 85 studies were excluded for not meeting the inclusion criteria, as detailed in the study selection flowchart (Fig. 1). In total, 65 RCTs were included in the final systematic review and 59 RCTs in the meta-analysis, involving a total of 2102 individuals (905 with and 1197 without T2D). The RCTs were classified according to the design of the study as acute post-prandial (*n* = 37, Tables 1, 2) or long-term (*n* = 28, Tables 3, 4) trials and separated into normoglycemic (Tables 1, 3) and T2DM (Tables 2, 4).

**Table 1:** Summary of acute RCTs investigating the effect of pulse intake on glycemic indices in normoglycemic adults

References	Country of study	Design	N	Age, y^1^	BMI^1^	Pulse type	Format	Other CHO source	Total CHO (pulse only), g	Control	Outcomes
Agustia et al. [43]	Indonesia	NR	11	20.1 ± 1.3	20.9 ± 1.9	Beans	Flour	Rice	50	Glucose	Glucose
Akhtar et al. [44]	Pakistan	C	24	22.5 ± 2.4	21.8 ± 1.7	Beans	Flour	Wheat flour	50 (20)	Wheat flour	Glucose, insulin
Anderson et al. [18]	Canada	C	17	22.1 ± 3.0	22.9 ± 1.2	Beans, lentils, chickpeas	Whole, pureed and Flour	Tomato sauce	38.7 (25)	Whole wheat flour	Glucose
Anguah et al. [20]	US	C	12	28.0 ± 10.0	23.3 ± 3.1	Lentil	Whole, pureed	Rice, wheat	NR	Rice and egg burritos	Glucose
Augustin et al. [21]	Canada	C	10	53.0 ± 7.0	29.4 ± 3.8	Chickpeas	Pureed	–	25	White bread	Glucose, insulin
Boers et al. [23]	UK	C	12	37 ± 9	22.8 ± 1.6	Chickpeas	Flour	Wheat	57 (8.5)	High-fiber flat bread	Glucose
Bornet et al. [25]	France	C	6	23.9 ± 1.7	20.6 ± 1.7	Beans	Flour	–	35	Extruded wheat	Glucose, insulin
Dandachy et al. [27]	Lebanon	C	16	22.9 ± 12	22.7 ± 10.6	Chickpeas	Flour	Wheat	NR	Wheat flour	Glucose
Dilwari et al. [29]	India	C	6	36.3 ± 9.7	NR	Lentils, beans	Whole	–	50	Rice	Glucose
Greffeuille et al. [45]	France	C	15	24 ± 11.2	22.4 ± 7.0	Beans	Flour	Wheat	50 (17.5)	Wheat pasta	Glucose, insulin
Jenkins et al. [33]	UK	C	10	NR	NR	Beans; peas; chickpeas; lentils	Whole	–	50	White bread	Glucose
Jenkins et al. [30]	UK	C	9	29.0 ± 8.0	NR	Lentils	Whole	–	50	White bread	Glucose
Johnson et al. [46]	Australia	C	11	32.0 ± 6.6	24.7 ± 2.7	Chickpeas	Flour	Jam, milk	50 (NR)	White bread	Glucose, insulin
Marinangeli et al. [47]	Canada	C	22	NR	NR	Peas	Flour, whole	Wheat	50 (NR)	White bread	Glucose
Mehio et al. [48]	Lebanon	C	12	24.0 ± 3.4	22.8 ± 2.1	Chickpeas	Pureed	NR	50	White bread	Glucose, insulin
Mollard et al. [49]	Canada	C	25	21.3 ± 2.5	21.6 ± 1.5	Chickpeas, lentils, peas	Whole	Macaroni	98.7 (40)	Macaroni and cheese	Glucose
Moravek et al. [50]	Canada	C	24	27.4 ± 1.2	24.3 ± 0.5	Lentils	Whole	Rice/potato	50 (NR)	Rice or potatoes	Glucose, insulin
Nestel et al. [51]	Australia	C	19	61.5 ± 6.4	26.5 ± 3.8	Chickpeas	Pureed	Milk	50 (33)	White bread and jam	Glucose
Potter et al. [52]	US	C	8	NR	NR	Beans	Pureed	–	75	Brown rice	Glucose
Ramdath et al. [53]	Canada	C	10	45.1 ± 11.0	27.7 ± 6.1	Lentils	Whole	–	25, 25	White bread	Glucose
Ramdath, et al. [6]	Canada	C	10	40.0 ± 10.0	25.0 ± 4.1	Lentils	Whole, pureed and Flour	–	50	Potatoes	Glucose
Reverri et al., (2015) [54]	US	C	12	49.0 ± 14.0	32.2 ± 5.7	Beans	Pureed	–	NR	Couscous	Glucose
Tappy et al. [55]	Switzerland	C	6	NR	NR	Beans	Flakes	–	50	Potatoes	Glucose
Torsdottir et al. [56]	Sweden	C	6	24.0 ± 6.0	22.2 ± 1.1	Beans	Pureed	–	43	Potatoes	Glucose, insulin
Traianedes et al. [57]	Australia	C	6	30.0 ± 10.0	24.3 ± 1.7	Beans	Whole	–	50	Glucose	Glucose, insulin
Winham et al. [58]	US	C	12	36.0 ± 15.0	23.3 ± 5.4	Black beans, chickpeas	Whole	Rice	50 (15)	Rice	Glucose, insulin
Wong et al. [59]	Canada	C	14	NR	NR	Beans, chickpeas, lentils, peas	Whole	–	50	White bread	Glucose
Yoshimoto et al. [60]	Japan	C	12	37.8 ± 9.5	22.9 ± 3.5	Peas	Flour	–	50	Rice	Glucose, insulin
Zafar et al. [61]	Kuwait	C	13	21.4 ± 2.3	23.6 ± 2.4	Chickpeas	Flour	Wheat, milk	NR	White bread	Glucose
Zhu et al. [62]	China	C	10	20.7 ± 2.3	22.0 ± 2.1	Beans	Whole	–	50	White rice	Glucose
Zurbau et al. [63]	Canada	C	21	26.7 ± 12.3	22.2 ± 2.8	Chickpeas	Whole	Tomatoes	50 (NR)	Potatoes	Glucose

^1^Age and BMI are reported as mean ± SD; *BMI* body mass index, *CHO* available carbohydrates, *C* crossover, *N* number of participants, *NR* not reported

**Table 2:** Summary of acute RCTs investigating the effect of pulse intake on glycemic indices in T2D adults

References	Country of study	Design	N	Age, y^1^	BMI^1^	Pulse type	Format	Other CHO source	Total CHO (pulse only), g	Control	Outcomes
Bornet et al. [24]	France	C	18	57 ± 8.5	27.9 ± 4.7	Lentils, beans	Whole	–	50	Glucose	Glucose, insulin
Jenkins et al. [31]	UK	C	6	43 ± 5	NR	Lentils	Whole	Soya	50 (23)	Whole meal bread	Glucose
Mani et al. [64]	India	C	6	58 ± 9	NR	Lentils	Whole	Semolina	50 (16)	Semolina	Glucose
Olmedilla-Alonso et al. [65]	Spain	C	12	66.4 ± 6.2	30.1 ± 3.6	Beans	Whole	–	57.8	White bread	Glucose, insulin
Schafer et al. [66]	Germany	C	9	61 ± 14	29.9 ± 8.7	Peas	Whole	Carrots	40 (37)	Potato	Glucose, insulin
Thompson et al. [67]	US	C	17	58.6 ± 20	31.9 ± 7.9	Beans	Whole	Rice	50 (15)	Rice only	Glucose

^1^Age and BMI are reported as mean ± SD; *BMI* body mass index, *CHO* available carbohydrates, *C* crossover, *N* number of participants, *NR* not reported

**Table 3:** Summary of long-term RCTs investigating the effect of pulse intake on glycemic indices in normoglycemic adults

References	Country of study	Design	Duration, weeks	N	Age, y^1^	BMI^1^	Intervention	Dose, g/day	Control	Outcomes
Abete et al. [14]	Spain	P	8	32	NR	32.5 ± 4.3	Low GI diet with pulse intake	130	Energy-restricted high GI diet	Glucose, insulin, HOMA-IR
Abete et al. [15]	Spain	P	8	35	38.0 ± 7.0	31.8 ± 3.0	High-pulse diet	100	Energy-restricted diet	Glucose
Abeysekara et al. [16]	Canada	C	8	87	59.7 ± 6.3	27.5 ± 4.5	Pulse-based diet	250	Regular diet	Glucose, insulin
Alizadeh et al. [17]	Iran	P	6	34	36.1 ± 8.2	NR	hypocaloric diet enriched in pulses	190	Hypocaloric diet	Glucose, insulin, HOMA-IR
Anderson et al. [19]	US	P	3	10	53.9 ± 8.5	NR	Beans supplemented diet	115	Oat bran diet	Glucose
Cryne et al. [26]	Canada	C	4	21	28.1 ± 5.9	25.2 ± 3.5	Spray-dried chickpeas, lentils, peas	100	Dehydrated potato flakes	Glucose, insulin, HOMA-IR
Gravel et al. [68]	Canada	P	16	132	51.7 ± 8.6	29.8 ± 5.1	Pulse-based meals	110	Isocaloric control meals	Glucose, insulin
Kim et al. [69]	Australia	C	4	51	35.1 ± 15.6	27.7 ± 6.9	Diet high in dairy, whole grains, nuts and pulses	150–225	Diet high in red and meat and refined grains	Glucose
Marinangeli et al. [70]	Canada	C	4	23	52.0 ± 11.2	30.5 ± 4.4	whole pea flour muffin	50	White wheat flour muffin	Glucose
Nestel et al. [51]	Australia	C	6	20	56.6 ± 7.6	25.6 ± 3.2	Chickpea based diet	200	Wheat-based diet	Glucose, insulin, HOMA-IR
Pittaway et al. [71]	Australia	C	5	27	50.6 ± 10.5	28.8 ± 4.4	Chickpeas based diet	200	Low fiber wheat-based diet	Glucose, insulin, HOMA-IR
Saraf-Bank et al. [72]	Iran	C	6	26	50.0 ± 6.6	28.9 ± 4.3	Habitual diet enriched with pulses	65	Habitual diet without pulses	Glucose, HbA_1c_
Tonstad et al. [73]	US	P	16	123	48.4 ± 10.7	36.4 ± 3.5	High-fiber bean-rich diet	125	Low-carbohydrate diet	Glucose, HbA_1c_
Tovar et al. [74]	Sweden	C	4	46	61.6 ± 5.4	28.8 ± 8.1	Whole grain, barley and pulse rich diet	168	Low pulse diet	Glucose, insulin, HbA_1c_, HOMA-IR
Venn et al. [75]	New Zealand	P	72	113	42.0 ± 10.7	35.4 ± 5.5	High pulse diet	180	Low pulse diet	
Winham et al. [76]	US	C	8	16	43.0 ± 20.0	27.8 ± 5.6	Beans/peas enriched diet	120	Carrot enriched diet	Glucose, insulin, HbA_1c_, HOMA-IR

^1^Age and BMI are reported as mean ± SD, *BMI* body mass index, *C* crossover, *N* number of participants, *NR* not reported, *P* parallel study design

**Table 4:** Summary of long-term RCTs investigating the effect of pulse intake on glycemic indices in T2D adults

References	Country of study	Design	Duration, weeks	N	Age, y^1^	BMI^1^	Intervention	Dose, g/day	Control	Outcomes
Hassanzadeh-Rostami et al. [77]	Iran	P	8	64	59.6 ± 5.9	27.3 ± 3.4	Pulses	100	Red meat	Glucose, insulin, HbA_1c_
Hosseinpour-Niazi et al. [78]	Iran	C	8	31	58.1 ± 6.0	27.7 ± 3.3	Pulse-based TLC diet	190	Pulse-free TLC diet	Glucose, insulin
Islam et al. [79]	Bangladesh	P	4	30	52.4 ± 5.6	25.1 ± 2.2	Mixed pulse and wheat bread	NR	Wheat bread	Glucose
Jang et al. [80]	Republic of Korea	P	16	76	56.6 ± 8.6	24.6 ± 2.2	Black bean powder mixed with wholegrains powder	15	Cooked refined rice	Glucose, insulin, HOMA-IR
Jenkins et al., (2012) [81]	Canada	P	12	121	53.0 ± 10.0	29.9 ± 5.5	Low GI pulse diet	190	High wheat fiber diet	Glucose, HbA_1c_
Jimenez-Cruz et al. [82]	US	C	6	14	53.0 ± 9.0	32.3 ± 5.9	Low GI Mexican style diet with pulses	35	High GI Mexican style diet	
Jimenez-Cruz et al. [83]	US	C	3	8	51.0 ± 3.0	30.7 ± 7.9	Low GI high fiber diet with pulse	NR	High GI low fiber diet	Glucose, HbA_1c_
Kang et al. [84]	Republic of Korea	P	12	185	50.4 ± 9.9	25.5 ± 3.2	Whole grains and pulses	30–70	Refined rice diet	Glucose, insulin, HOMA-IR
Kim et al. [5]	Republic of Korea	P	12	99	55.4 ± 11.9	24.1 ± 3.4	Whole grains and pulses	30–70	Refined rice diet	Glucose, insulin, HbA_1c_, HOMA-IR
Kim et al. [85]	Republic of Korea	P	12	80	NR	NR	Whole grains and pulses	30–70	Refined rice diet	Glucose, insulin, HbA_1c_, HOMA-IR
Liu et al. [86]	China	P	4	106	57.4 ± 8.8	26.6 ± 1.0	Extruded adzuki bean convenient food	170	Low GI diet	Glucose, insulin, HbA_1c_
Winham et al. [87]	US	C	8	23	45.9 ± 21	27.4 ± 5.1	Canned baked navy beans	130	Canned carrots	Glucose, insulin, HbA_1c_, HOMA-IR

^1^Age and BMI are reported as mean ± SD; *BMI* body mass index, *C* crossover, *N* number of participants, *NR* not reported

Assessment of risk of bias across the studies indicated concerns for the majority of RCTs due to lack of information on randomization concealment as well as selection of the reported results (Supplemental Table 2). There were ten RCTs that fell into the ‘high risk’ category due to concerns in three or more domains. These were mainly the trials that were published more than 20 years ago; in appreciation of the fact that the standards on reporting RCTs were substantially different then, we have not removed these studies from the meta-analysis.

### Parameters of post-prandial glycemic control

**Fig. 2:**
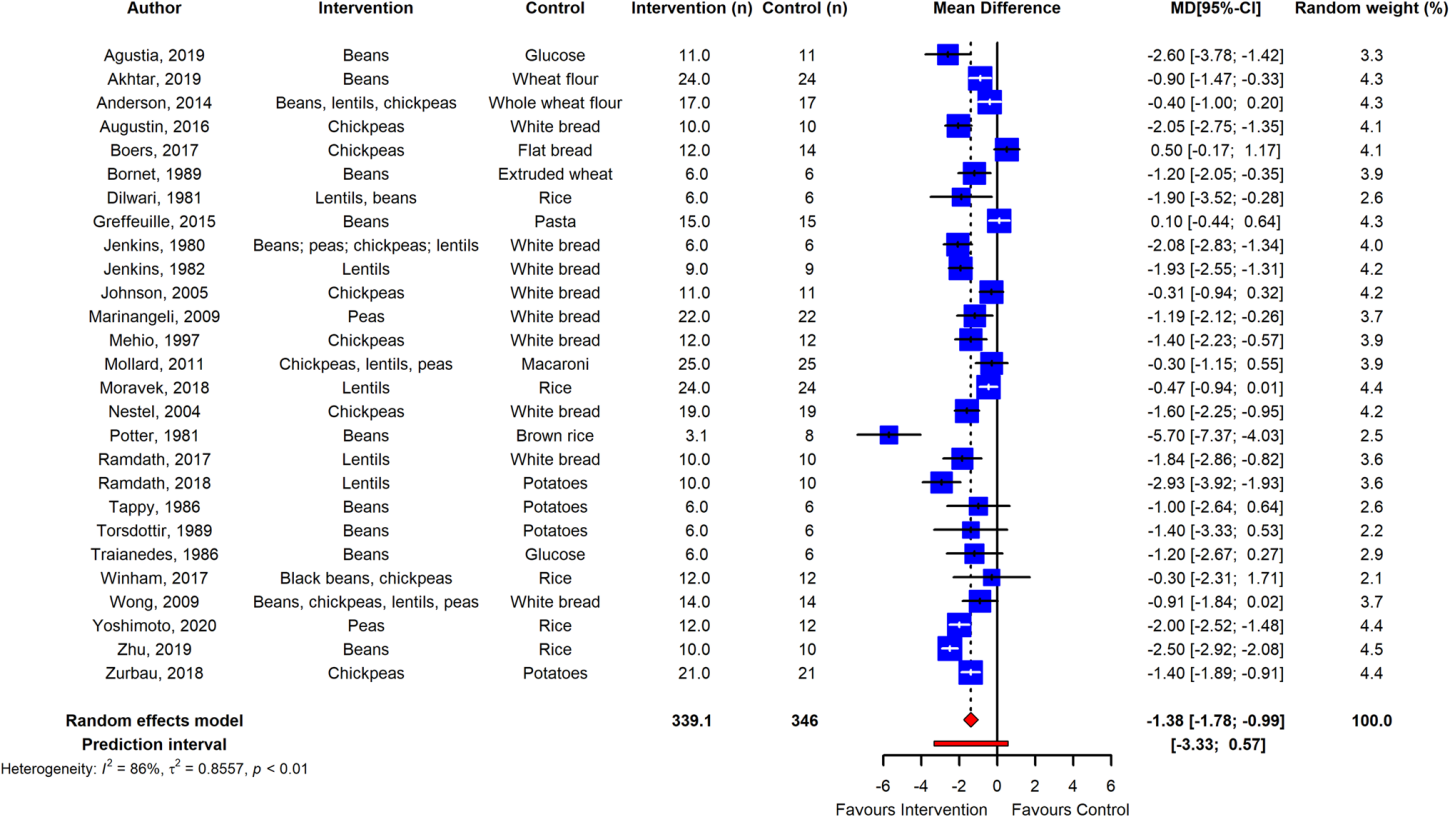
Pooled effect using inverse-variance random effect model (mean difference and 95% CI) of acute trials investigating pulse intake on post-prandial glucose response among healthy individuals. The effect size was statistically significant for normoglycemic adults

**Fig. 3:**
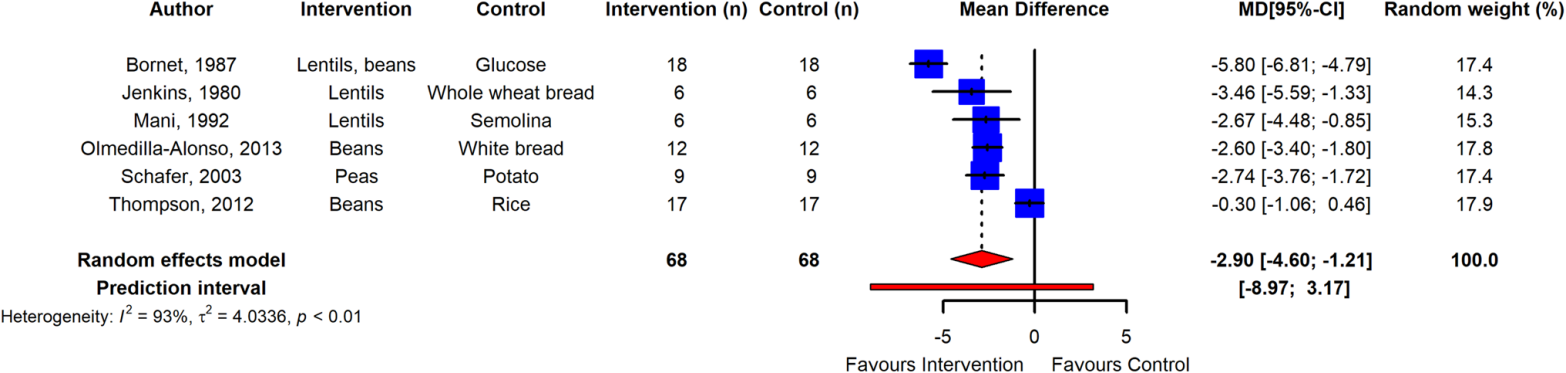
Pooled effect using inverse-variance random effect model (mean difference and 95% CI) of acute trials investigating pulse intake on post-prandial glucose response among T2D individuals. The effect size was statistically significant for adults with T2D

The meta-analysis showed that pulse intake significantly improved parameters of post-prandial glycemic handling. Post-prandial plasma glucose was overall significantly reduced in normoglycemic adults (*n* = 27 RCTs, ES  – 1.38; 95% CI  – 1.78,  – 0.99; *p* ≤ 0.001; *I*^2^ = 86%, PI  – 3.33, 0.57) and in adults with T2D (*n* = 6 RCTs, ES  – 2.90; 95% CI  – 4.60,  – 1.21; *p* ≤ 0.001; *I*^2^ = 93%, PI  – 8.97, 3.17) (Figs. 2, 3), with high heterogeneity between studies. Egger’s test of publication bias did not indicate presence of funnel plot asymmetry (*p* > 0.05) (Supplemental Fig. 1). Subgroup analysis of pulse type revealed that lentils (*n* = 9 RCTs) were most effective in reducing PPGR (ES  – 1.60; 95% CI  – 2.23,  – 0.97; *p* ≤ 0.0001, *I*^2^ = 84%), followed by dried peas (*n* = 5 RCTs; ES  – 1.32; 95% CI  – 2.07,  – 0.56; *p* ≤ 0.005, *I*^2^ = 81%), beans (*n* = 14 RCTs; ES  – 1.18; 95% CI  – 1.74,  – 0.62; *p* < 0.0001, *I*^2^ = 82%), and chickpeas (*n* = 11 RCTs; ES  – 0.97; 95% CI  – 1.48,  – 0.47; *p* < 0.001, *I*^2^ = 78%). However, the differences in ES were not significant between types of pulses (*p* = 0.49) (Supplemental Fig. 2). Furthermore, analysis and meta-regression of processing method revealed that ES was significantly lower when pulse flour was used as intervention (*n* = 10 RCTs; ES  – 0.81; 95% CI  – 1.33,  – 0.29; *p* ≤ 0.005, I^2^ = 83%) compared to whole (*n* = 14 RCTs; ES  – 1.84; 95% CI  – 2.32,  – 1.37; *p* ≤ 0.0001, *I*^2^ = 80%) and pureed pulse (*n* = 7 RCTs; ES  – 1.65; 95% CI  – 2.33,  – 0.98; *p* ≤ 0.0001, *I*^2^ = 70%) with (*p* < 0.05) for subgroup differences (Supplemental Fig. 3). Moreover, subgroup analysis by grouping control foods used in the post-prandial trials suggested that the ES was greater when potatoes were used as control and pasta was the lowest (Supplemental Fig. 4). Sensitivity analysis by removal of studies with high risk of bias did not change the ES.

**Fig. 4:**
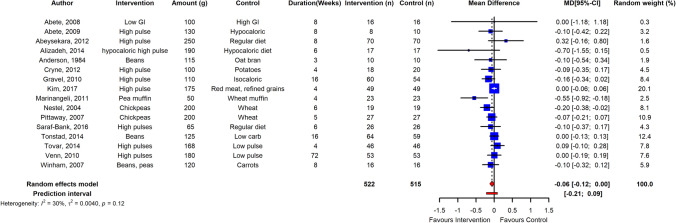
Pooled effect using inverse-variance random effect model (mean difference and 95% CI) of long-term trials investigating pulse intake on fasting glucose among healthy individuals. The meta-analysis concluded that long-term pulse intake has small but significant effect on reducing fasting blood glucose levels in normoglycemic adults

ES of post-prandial insulin responses were also significantly lower in both adults with and without T2DM (*n* = 3 RCTs; ES  – 19.43; 95% CI  – 24.01,  – 14.85; *p* ≤ 0.0001, *I*^2^ = 0%) and (*n* = 11 RCTs; ES  – 11.26; 95% CI  – 22.11,  – 0.41; *p* ≤ 0.05, *I*^2^ = 90%), respectively.

### Long-term parameters of glycemic control

The meta-analysis revealed that long-term pulse intake has a small reducing effect on fasting blood glucose levels in normoglycemic adults (n = 16 RCTs) with low heterogeneity between studies (ES  – 0.06; 95% CI  – 0.12, 0.00; *p* ≤ 0.05; *I*^2^ = 30%; PI  – 0.21, 0.09) (Fig. 4). Sensitivity analysis showed that independent removal of one trial changed the ES interpretation from significant to non-significant. Pulse consumption in normoglycemic adults had no significant effect on fasting insulin, HbA_1c_ and HOMA-IR, although the effect direction was toward reduction (*n* = 9 RCTs; ES  – 0.11; 95% CI  – 0.76, 0.55; *p* = 0.75); (*n* = 4 RCTs; ES  – 0.03; 95% CI  – 0.11, 0.06; *p* = 0.54); (*n* = 7 RCTs; ES  – 0.02; 95% CI  – 0.18, 0.14; *p* = 0.78), respectively.

**Fig. 5:**
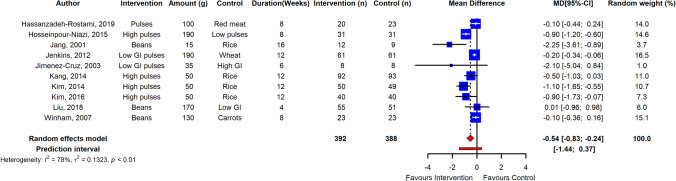
Pooled effect using inverse-variance random effect model (mean difference and 95% CI) of long-term trials investigating pulse intake on fasting glucose among T2D individuals. Long-term pulse intake resulted in a significant reduction of fasting blood glucose in adults with T2D

**Fig. 6:**
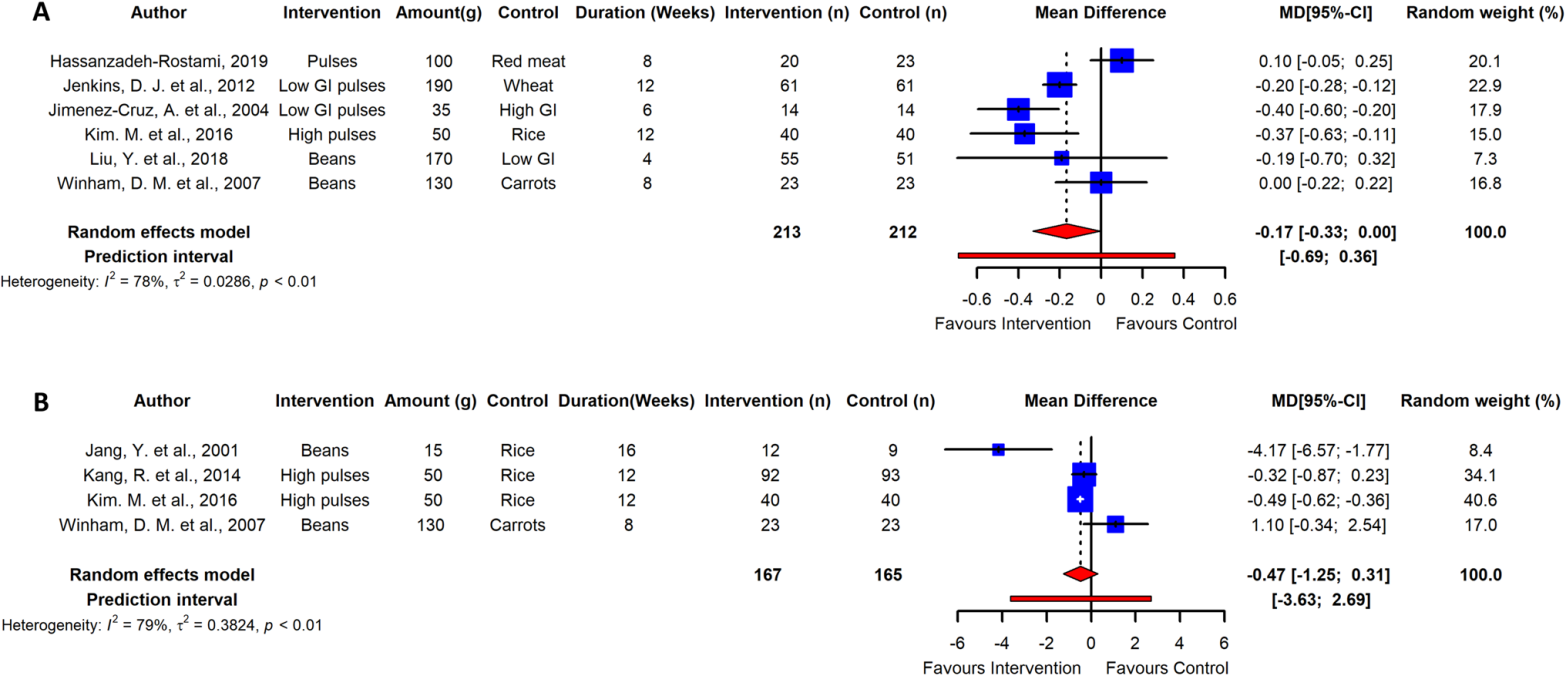
Pooled effect using inverse-variance random effect model (mean difference and 95% CI) of long-term trials investigating pulse intake on fasting glycated hemoglobin (**a**); and HOMA-IR (**b**) among T2D individuals

Long-term pulse intake resulted in a significant reduction of fasting blood glucose in adults with T2D as estimated from data of 10 RCTs (ES  – 0.54; 95% CI  – 0.83,  – 0.24; *p* ≤ 0.005; *I*^2^ = 78%; PI  – 1.44, 0.37), albeit with high heterogeneity among studies (Fig. 5). HbA_1c_ and HOMA-IR were also significantly reduced in adults with T2DM with high heterogeneity between studies (*n* = 6 RCTs; ES  – 0.17; 95% CI  – 0.33,  – 0.00; *p* ≤ 0.05; *I*^2^ = 78; PI  – 0.69, 0.36) and (*n* = 4 RCTs; ES  – 0.47; 95% CI  – 1.25,  – 0.31; *p* ≤ 0.05; *I*^2^ = 79%; PI  – 3.63, 2.69) (Fig. 6). Sensitivity analysis revealed that independent removal of one trial in estimation of ES of HbA_1c_ reduced the heterogeneity significantly [77], and removal of two RCTs changed the interpretation from significant to non-significant when estimating the ES of HOMA-IR [5, 85]. However, reduction in fasting blood insulin in T2DM adults was not significant (*n* = 8 RCTs, ES  – 1.18; 95% CI  – 2.54,  – 0.08; *p* > 0.05; *I*^2^ = 63%). Egger’s test did not indicate funnel plot asymmetry in long-term trials (*p* > 0.05) (Supplemental Figs. 5, 6).

The GRADE assessment for each outcome, summarized in Supplemental Table 3, revealed ‘low’ grades for acute PPGR in normoglycemic and T2DM, mainly downgraded due to inconsistency and indirectness of these outcomes. Evidence on long-term parameters fasting glucose, HbA_1c_ were graded as ‘very low’ due to low ratings for consistency, directness, and precision that led to decrease in the level of certainty.

## Discussion

In this systematic review and meta-analysis, we found that pulse intake enhances glycemic regulation on both acute post-prandial responses and long-term glycemic indices. We demonstrate that pulse intake leads to clinically significant reductions in PPGRs, with a mean reduction of PPGR > 1 mmol/L in normoglycemic individuals, and > 2.5 mmol/L in those with T2D, and consequently significantly reduced insulin was observed ≥ 20 mIU/L. Long-term pulse intake was reported to reduce fasting glucose, HbA_1c_ and HOMA-IR with more pronounced effect in adults with T2DM.

Post-prandial glycemic control plays a crucial role in prevention of chronic diseases such as cardiovascular disease, in both normoglycemic and T2D individuals [88]. The estimated magnitude of the reduction in PPGR is similar to the reported effect of some glucose lowering therapies such as DPP-4 inhibitors [89, 90]. However, the certainty of evidence is impaired due to substantial inter-study variances. Possible modifiers were identified in acute RCTs, such as differences in pulse type, processing methods, and the control used as a comparison, which were explored by subgroup analysis. Although lentils are suggested by subgroup analysis to be the most potent type in controlling PPGR, other types of pulses still show a clinically significant impact (range  – 1.60 to  – 0.95 mmol/L) in normoglycemic adults with substantial inter-study heterogeneity. There are only a few trials that have assessed the impact of processing on post-prandial glycemic responses and the results are mixed with some RCTs finding no significant impact of processing in attenuating PPGR, while others suggest that pulse flour resulted in significantly higher PPGR in comparison to other physical forms [6, 18, 20]. Our meta-analysis supports the finding that intervention foods using pulse flour were found to be 50% less effective in attenuating PPGR when comparing to other physical forms. However, pulse flour used as intervention in the RCTs was incorporated into bakery products or pasta, with the flour being only 25–35% of the composition of final product; the incorporation of legume flour with cereal flours resulted in a lower effect when compared to whole pulses which were mostly consumed alone. Nevertheless, the lower efficacy of pulse flour could also be explained by breakage of the cell walls during the milling process, resulting in increased exposure of the starch to digestive enzymes whilst wet pureeing may result in cell separation, keeping more cells intact [6]. However, due to the high heterogeneity within subgroups, possibly due to the presence of different pulse types within a subgroup and lack of standardized protocol for food processing, definitive outcomes cannot be concluded and, therefore, more studies are needed to investigate effects of processing on post-prandial glycemic handling.

In alignment with blood glucose, pulse intake favorably affected post-prandial insulin levels with a larger effect in T2D population where reduction in PPGR was greater. There were large variations between RCTs with regards to characteristics of participants such as mean age (22–66 y) and BMI (20–31), that might influence insulin secretion and sensitivity.

Long-term RCTs show that pulse intake leads to a favorable impact on fasting blood glucose in adults with and without T2D, and improved HbA_1c_ and HOMA-IR in those with T2D. The attenuation of fasting blood glucose was small in normoglycemic individuals (mean difference of ⁓0.06 mmol/L over median duration of 6 weeks), and greater in with T2D (mean difference of ⁓0.5 mmol/L over median duration of 8 weeks). We conducted a comparison of ES considering presence of diabetes as a modifier, and found significant differences between both conditions (*p* < 0.05) (Supplemental Fig. 7).

Post hoc meta-regression was performed to investigate the effect of pulse dose and study duration, and found low doses of pulses were more effective in reducing fasting blood glucose in adults without T2DM. However, there was no significant effect of study duration in modifying the ES (Supplemental Figs. 8 and 9). Our findings are in agreement with Sievenpiper et al. reporting inverse association between pulse dose in interventions and ES [35].

The reduction of HbA_1c_ (mean reduction of ⁓ 0.3%) is also considered to be clinically significant as the effect is comparable to low doses of some oral anti-diabetic agents such as α-glucosidase inhibitors [91]. Considering that HbA_1c_ reflects average glucose levels over the 8–12-week life span of erythrocytes [92], it is not surprising that some studies with an intervention duration shorter than this did not report an improvement in this measure. This together with subgroup analysis of study duration emphasizes the importance of conducting long-term RCTs of > 8 weeks in duration to report the outcomes of pulse intake and other dietary interventions on measures of glycemic control.

The beneficial effect of pulse intake on regulation of glucose metabolism could be related to several mechanisms. The bioavailability of carbohydrates from pulses can be reduced by factors such as low free sugar content and high levels of resistant starch [13]. In cooked whole or blended pulses, the presence of thick cell walls is likely to prevent access of amylolytic enzymes to the starch substrate [93]. Thermal processing increases fiber solubility, but the impact of this on glycemic effects is not known [94]. Furthermore, the crystalline nature of pulse starch and presence of fiber polysaccharides (both soluble and insoluble) as well as protein and lipids, contribute to delaying the gastric transit thereby slowing the arrival of food into the small intestine and hence lower the glycemic response [13, 95].

Other systemic effects may be via the microbial fermentation of fiber and resistant starch in the colon to short-chain fatty acids (SCFA) such as propionate, butyrate and acetate [96]. These SCFA reduce glucose release from the liver and thus promote muscle glycolysis, improved insulin secretion and glucose homeostasis via gut-brain axis and suppression of free fatty acid synthesis [97]. The soluble fiber is suggested to have beneficial impact on reduction of post-prandial glycemic effects attributing to the viscosity and gel-forming properties [98, 99]. Presence of fiber along with slowly digestible starch in pulses has been linked to improved blood glucose profile, insulin sensitivity and urinary C-peptide, and tends to normalize insulin levels in individuals with hyperinsulinemia [100].

To our knowledge, this is the first meta-analysis summarizing the impact of pulse intake on acute PPGR reported after pulse intake, and the most comprehensively assessing long-term impact of pulse consumption on glycemic handling indices. Post-prandial glycemic biomarkers are highly correlated with long-term indices and are considered as independent risk factors in progression of several health conditions such as diabetes and coronary heart diseases [101]. Therefore, including acute post-prandial trials in this review, and adopting raw mean difference over standardized mean difference beside employment of meta-regression allow better understanding over previous meta-analysis regarding the role of pulses in controlling glycemic indices [35]. Furthermore, we have assessed the certainty of the evidence by following GRADE method, and calculated the prediction intervals to estimate clinical consequences of the heterogeneity and to provide a range into which we can predict the outcome of future studies to fall based on current evidence. Our prediction intervals are broad including both positive and negative intervals, reducing the confidence in predicting that results of a future trial would favor pulse intake, although broad prediction intervals are common in RCTs. However, there are several limitations in our analysis that should be considered. First, the risk of bias ranged from ‘some concerns’ to ‘high risk’, and the quality of evidence was graded from ‘low to ‘very low’. This is largely due to substantial inter-study heterogeneity that remained unexplained despite subgroup analysis. Additional variables such as ethnic background, genetic predisposition, physiological factors such as age, gender and BMI, lifestyle of the participants might contribute toward observed heterogeneity in reported outcomes and thus affecting the grading of the evidence. The quality of the RCTs was downgraded mostly due to inappropriate way in conducting or reporting of randomization process, or due to unavailability of trial protocol or register information. These factors collectively reinforce importance of high quality RCTs to support the beneficial effect of pulse intake on glycemic handling [102]. Second, we have included only RCTs with defined pulse consumption in the meta-analysis while excluding those that included pulses in selective eating patterns such as low GI or high-fiber diets. While this may have reduced the number of studies included, it also increased knowledge about particular types and forms of pulses. Third, there were 28 studies excluded due to inability of accessing the full text, and 3 papers were excluded as they were not available in English language. These collectively might have resulted in publication bias. Finally, the data extraction procedure was performed by single author which might introduced some biases.

Overall, pulse intake significantly reduced PPGR in both normoglycemic and individuals with T2D and, therefore, are recommended for consumption as a low GI food. Long-term pulse consumption resulted in favorable effects on measures of glycemic control especially in those with T2D. Although whole or pureed lentils showed more promising effects, due to high heterogeneity between studies, it is not possible to give a specific recommendation with regards to pulse type, dose, form (i.e. processing method) and duration of intake. Carefully controlled acute studies are required to study the impact of differently processed pulses on glycemic parameters. Furthermore, well-designed long-term RCTs are needed to establish effectiveness of pulse rich diets and dose–response relationships to refine dietary recommendations for pulse intake.

## Supplementary Information

Below is the link to the electronic supplementary material.

Supplementary file1 (DOC 335 KB)

## Abbreviations

ADA: American diabetes association
AUC: Area under the curve
CHO: Carbohydrates
CI: Confidence interval
EASD: European association for the study of diabetes
ES: Effect size
GI: Glycemic index
HbA_1c_: Glycated hemoglobin A1c
HOMA-IR: Homeostatic model assessment of insulin resistance
PPGR: Post-prandial glucose response
PI: Prediction intervals
RCTs: Randomized controlled trials
SCFA: Short-chain fatty acids
T2D: Type 2 diabetes

## References

[1] Rydén L, Grant PJ, Anker SD, Berne C, Cosentino F, Danchin N, Deaton C, Escaned J (2013). ESC Guidelines on diabetes, pre-diabetes, and cardiovascular diseases developed in collaboration with the EASD: the task Force on diabetes, pre-diabetes, and cardiovascular diseases of the European Society of Cardiology (ESC) and developed in collaboration with the European Association for the Study of Diabetes (EASD). Eur Heart J.

[2] Evert AB, Dennison M, Gardner CD, Garvey WT, Lau KHK, MacLeod J, Mitri J, Pereira RF, Rawlings K, Robinson S (2019). Nutrition therapy for adults with diabetes or prediabetes: a consensus report. Diabetes Care.

[3] Agrawal S, Ebrahim S (2013). Association between legume intake and self-reported diabetes among adult men and women in India. BMC Public Health.

[4] Villegas R, Gao Y-T, Yang G, Li H-L, Elasy TA, Zheng W, Shu XO (2008). Legume and soy food intake and the incidence of type 2 diabetes in the Shanghai Women's Health Study. Am J Clin Nutr.

[5] Kim M, Jeung SR, Jeong TS, Lee SH, Lee JH (2014). Replacing with whole grains and legumes reduces Lp-PLA2 activities in plasma and PBMCs in patients with prediabetes or T2D. J Lipid Res.

[6] Ramdath D, Wolever T, Siow Y, Ryland D, Hawke A, Taylor C, Zahradka P, Aliani MJF (2018). Effect of processing on postprandial glycemic response and consumer acceptability of lentil-containing food items. Foods.

[7] Singh N (2017). Pulses: an overview. J Food Sci Technol.

[8] Brummer Y, Kaviani M, Tosh SM (2015). Structural and functional characteristics of dietary fibre in beans, lentils, peas and chickpeas. Food Res Int.

[9] Fabbri ADT, Schacht RW, Crosby GA (2016). Evaluation of resistant starch content of cooked black beans, pinto beans, and chickpeas. NFS J.

[10] King DG, Walker M, Campbell MD, Breen L, Stevenson EJ, West DJ (2018). A small dose of whey protein co-ingested with mixed-macronutrient breakfast and lunch meals improves postprandial glycemia and suppresses appetite in men with type 2 diabetes: a randomized controlled trial. Am J Clin Nutr.

[11] Padhi EMT, Ramdath DD (2017). A review of the relationship between pulse consumption and reduction of cardiovascular disease risk factors. J Funct Foods.

[12] Prasad V, Adapa D, Vana D, Choudhury A, Asadutullah J, Chatterjee A (2018). Nutritional components relevant to type-2-diabetes: dietary sources, metabolic functions and glycaemic effects. J Res Med Dent Sci.

[13] Perez-Hernandez LM, Nugraheni K, Benohoud M, Sun W, Hernández-Álvarez AJ, Morgan MR, Boesch C, Orfila C (2020). Starch digestion enhances bioaccessibility of anti-inflammatory polyphenols from borlotti beans (*Phaseolus vulgaris*). Nutrients.

[14] Abete I, Parra D, Martinez JA (2008). Energy-restricted diets based on a distinct food selection affecting the glycemic index induce different weight loss and oxidative response. Clin Nutr.

[15] Abete I, Parra D, Martinez JA (2009). Legume-, fish-, or high-protein-based hypocaloric diets: effects on weight loss and mitochondrial oxidation in obese men. J Med Food.

[16] Abeysekara S, Chilibeck PD, Vatanparast H, Zello GA (2012). A pulse-based diet is effective for reducing total and LDL-cholesterol in older adults. Br J Nutr.

[17] Alizadeh M, Gharaaghaji R, Pourghassem Gargari B (2014). The effects of legumes on metabolic features, insulin resistance and hepatic function tests in women with central obesity: a randomized controlled trial. Int J Prev Med.

[18] Anderson G, Liu Y, Liu Y, Smith CE, Liu TT, Nunez MF, Mollard RC, Luhovyy BL (2014). The acute effect of commercially available pulse powders on postprandial glycaemic response in healthy young men. Br J Nutr.

[19] Anderson JW, Story L, Sieling B, Chen WJ, Petro MS, Story J (1984). Hypocholesterolemic effects of oat-bran or bean intake for hypercholesterolemic men. Am J Clin Nutr.

[20] Anguah KO, Wonnell BS, Campbell WW, McCabe GP, McCrory MA (2014). A blended- rather than whole-lentil meal with or without α-galactosidase mildly increases healthy adults' appetite but not their glycemic response. J Nutr.

[21] Augustin LSA, Chiavaroli L, Campbell J, Ezatagha A, Jenkins AL, Esfahani A, Kendall CWC (2016). Post-prandial glucose and insulin responses of hummus alone or combined with a carbohydrate food: a dose-response study. Nutr J.

[22] Barnard ND, Cohen J, Jenkins DJA, Turner-McGrievy G, Gloede L, Jaster B, Seidl K, Green AA, Talpers S (2006). A low-fat vegan diet improves glycemic control and cardiovascular risk factors in a randomized clinical trial in individuals with type 2 diabetes. Diabetes Care.

[23] Boers HM, MacAulay K, Murray P, Seijen Ten Hoorn J, Hoogenraad AR, Peters HPF, Vente-Spreeuwenberg MAM, Mela DJ (2017). Efficacy of different fibres and flour mixes in South-Asian flatbreads for reducing post-prandial glucose responses in healthy adults. Eur J Nutr.

[24] Bornet FR, Costagliola D, Rizkalla SW, Blayo A, Fontvieille AM, Haardt MJ, Letanoux M, Tchobroutsky G, Slama G (1987). Insulinemic and glycemic indexes of six starch-rich foods taken alone and in a mixed meal by type 2 diabetics. Am J Clin Nutr.

[25] Bornet FR, Fontvieille AM, Rizkalla S, Colonna P, Blayo A, Mercier C, Slama G (1989). Insulin and glycemic responses in healthy humans to native starches processed in different ways: correlation with in vitro alpha-amylase hydrolysis. Am J Clin Nutr.

[26] Cryne CN, Veenstra JM, Deschambault BR, Benali M, Marcotte M, Boye JI, Tosh SM, Farnworth ER, Wright AJ, Duncan AM (2012). Spray-dried pulse consumption does not affect cardiovascular disease risk or glycemic control in healthy males. Food Res Int.

[27] Dandachy S, Mawlawi H, Chedid M, El-Mallah C, Obeid OJF (2018). Impact of pre-processed chickpea flour incorporation into “mankoushe” on appetite hormones and scores. Foods.

[28] De Natale C, Annuzzi G, Bozzetto L, Mazzarella R, Costabile G, Ciano O, Riccardi G, Rivellese AA (2009). Effects of a plant-based high-carbohydrate/high-fiber diet versus high-monounsaturated fat/low-carbohydrate diet on postprandial lipids in type 2 diabetic patients. Diabetes Care.

[29] Dilawari JB, Kamath PS, Batta RP, Mukewar S, Raghavan S (1981). Reduction of postprandial plasma glucose by Bengal gram dal (Cicer arietinum) and rajmah (Phaseolus vulgaris). Am J Clin Nutr.

[30] Jenkins DJ, Thorne MJ, Camelon K, Jenkins A, Rao AV, Taylor RH, Thompson LU, Kalmusky J, Reichert R, Francis T (1982). Effect of processing on digestibility and the blood glucose response: a study of lentils. Am J Clin Nutr.

[31] Jenkins DJ, Wolever TM, Taylor RH, Ghafari H, Jenkins AL, Barker H, Jenkins MJ (1980). Rate of digestion of foods and postprandial glycaemia in normal and diabetic subjects. BMJ.

[32] Jenkins DJ, Wolever TM, Taylor RH, Griffiths C, Krzeminska K, Lawrie JA, Bennett CM, Goff DV, Sarson DL, Bloom SR (1982). Slow release dietary carbohydrate improves second meal tolerance. Am J Clin Nutr.

[33] Jenkins DJA, Wolever TMS, Taylor RH (1980). Exceptionally low blood glucose response to dried beans: comparison with other carbohydrate foods. BMJ.

[34] Viguiliouk E, Mejia SB, Kendall CW, Sievenpiper JL (2017). Can pulses play a role in improving cardiometabolic health? Evidence from systematic reviews and meta-analyses. Ann N Y Acad Sci.

[35] Sievenpiper J, Kendall C, Esfahani A, Wong J, Carleton A, Jiang H, Bazinet R, Vidgen E, Jenkins D (2009). Effect of non-oil-seed pulses on glycaemic control: a systematic review and meta-analysis of randomised controlled experimental trials in people with and without diabetes. Diabetologia.

[36] Liberati A, Altman DG, Tetzlaff J, Mulrow C, Gøtzsche PC, Ioannidis JPA, Clarke M, Devereaux PJ, Kleijnen J, Moher D (2009). The PRISMA statement for reporting systematic reviews and meta-analyses of studies that evaluate health care interventions: explanation and elaboration. Ann Intern Med.

[37] Grunberger G, Forst T, Fernández Landó L, Pechtner V, Shaginian R, Jia N, Gough S (2016). Early fasting glucose measurements can predict later glycaemic response to once weekly dulaglutide. Diabet Med.

[38] Sterne JA, Savović J, Page MJ, Elbers RG, Blencowe NS, Boutron I, Cates CJ, Cheng H-Y, Corbett MS, Eldridge SM (2019) RoB 2: a revised tool for assessing risk of bias in randomised trials. BMJ 366 10.1136/bmj.l4898 31462531

[39] Egger M, Smith GD, Schneider M, Minder C (1997). Bias in meta-analysis detected by a simple, graphical test. BMJ.

[40] Borenstein M, Hedges LV, Higgins JP, Rothstein HR (2011). Introduction to meta-analysis.

[41] Higgins JP, Thomas J, Chandler J, Cumpston M, Li T, Page MJ, Welch VA, Ed(s) (2021) Cochrane handbook for systematic reviews of interventions Version 6.2 (updated February 2021). Cochrane, 2021. Available from training.cochrane.org/handbook

[42] Inc EP, McMasterUniversity (2020). GRADEpro GDT: GRADEpro Guideline Development Tool [Software].

[43] Agustia FC, Subardjo YP, Ramadhan GR, Betaditya D (2019). Glycemic index of flakes made from Mocaf-Black rice and bean flour as alternative snacks for people with type 2 diabetes mellitus. Ann Trop Med Public Health.

[44] Akhtar S, Layla A, Sestili P, Ismail T, Afzal K, Rizvanov AA, Asad MHHB (2019). Glycemic and insulinemic responses of vegetables and beans powders supplemented chapattis in healthy humans: a randomized Crossover Trial. Biomed Res Int.

[45] Greffeuille V, Marsset-Baglieri A, Molinari N, Cassan D, Sutra T, Avignon A, Micard V (2015). Enrichment of pasta with faba bean does not impact glycemic or insulin response but can enhance satiety feeling and digestive comfort when dried at very high temperature. Food Funct.

[46] Johnson SK, Thomas SJ, Hall RS (2005). Palatability and glucose, insulin and satiety responses of chickpea flour and extruded chickpea flour bread eaten as part of a breakfast. Eur J Clin Nutr.

[47] Marinangeli CP, Kassis AN, Jones PJ (2009). Glycemic responses and sensory characteristics of whole yellow pea flour added to novel functional foods. J Food Sci.

[48] Mehio Z, Baba NH, Habbal Z (1997). Glycemic and insulinemic responses of normal subjects to selected meals commonly consumed in the Middle East. J Nutr Environ Med.

[49] Mollard RC, Wong CL, Luhovyy BL, Anderson GH (2011). First and second meal effects of pulses on blood glucose, appetite, and food intake at a later meal. Appl Physiol Nutr Metab.

[50] Moravek D, Duncan AM, VanderSluis LB, Turkstra SJ, Rogers EJ, Wilson JM, Hawke A, Ramdath DD (2018). Carbohydrate replacement of rice or potato with lentils reduces the postprandial glycemic response in healthy adults in an acute, randomized. Crossover Trial J Nutr.

[51] Nestel P, Cehun M, Chronopoulos A (2004). Effects of long-term consumption and single meals of chickpeas on plasma glucose, insulin, and triacylglycerol concentrations. Am J Clin Nutr.

[52] Potter JG, Coffman KP, Reid RL, Krall JM, Albrink MJ (1981). Effect of test meals of varying dietary fiber content on plasma insulin and glucose response. Am J Clin Nutr.

[53] Ramdath DD, Liu Q, Donner E, Hawke A, Kalinga D, Winberg J, Wolever TMS (2017). Investigating the relationship between lentil carbohydrate fractions and in vivo postprandial blood glucose response by use of the natural variation in starch fractions among 20 lentil varieties. Food Funct.

[54] Reverri EJ, Randolph JM, Steinberg FM, Kappagoda CT, Edirisinghe I, Burton-Freeman BM (2015). Black beans, fiber, and antioxidant capacity pilot study: examination of whole foods vs. functional components on postprandial metabolic, oxidative stress, and inflammation in adults with metabolic syndrome. Nutrients.

[55] Tappy L, Wursch P, Randin JP (1986). Metabolic effect of pre-cooked instant preparations of bean and potato in normal and in diabetic subjects. Am J Clin Nutr.

[56] Torsdottir I, Alpsten M, Andersson H, Schweizer TF, Tölli J, Würsch P (1989). Gastric emptying and glycemic response after ingestion of mashed bean or potato flakes in composite meals. Am J Clin Nutr.

[57] Traianedes K, O'Dea K (1986). Commercial canning increases the digestibility of beans in vitro and postprandial metabolic responses to them in vivo. Am J Clin Nutr.

[58] Winham DM, Hutchins AM, Thompson SV (2017). Glycemic response to black beans and chickpeas as part of a rice meal: a randomized cross-over trial. Nutrients.

[59] Wong CL, Mollard RC, Zafar TA, Luhovyy BL, Anderson GH (2009). Food intake and satiety following a serving of pulses in young men: effect of processing, recipe, and pulse variety. J Am Coll Nutr.

[60] Yoshimoto J, Kato Y, Ban M, Kishi M, Horie H, Yamada C, Nishizaki Y (2020). Palatable noodles as a functional staple food made exclusively from yellow peas suppressed rapid postprandial glucose increase. Nutrients.

[61] Zafar TA, Al-Hassawi F, Al-Khulaifi F, Al-Rayyes G, Waslien C, Huffman FG (2015). Organoleptic and glycemic properties of chickpea-wheat composite breads. J Food Sci Technol.

[62] Zhu R, Fan Z, Han Y, Li S, Li G, Wang L, Ye T, Zhao W (2019). Acute effects of three cooked non-cereal starchy foods on postprandial glycemic responses and in vitro carbohydrate digestion in comparison with whole grains: a randomized trial. Nutrients.

[63] Zurbau A, Jenkins AL, Jovanovski E, Au-Yeung F, Bateman EA, Brissette C, Wolever TMS, Hanna A, Vuksan V (2018). Acute effect of equicaloric meals varying in glycemic index and glycemic load on arterial stiffness and glycemia in healthy adults: a randomized crossover trial. Eur J Clin Nutr.

[64] Mani UV, Pradhan SN, Mehta NC, Thakur DM, Iyer U, Mani I (1992). Glycaemic index of conventional carbohydrate meals. Br J Nutr.

[65] Olmedilla-Alonso B, Pedrosa MM, Cuadrado C, Brito M, Asensio-S-Manzanera C, Asensio-Vegas C (2013). Composition of two Spanish common dry beans (Phaseolus vulgaris), 'Almonga' and 'Curruquilla', and their postprandial effect in type 2 diabetics. J Sci Food Agric.

[66] Schafer G, Schenk U, Ritzel U, Ramadori G, Leonhardt U (2003). Comparison of the effects of dried peas with those of potatoes in mixed meals on postprandial glucose and insulin concentrations in patients with type 2 diabetes. Am J Clin Nutr.

[67] Thompson SV, Winham DM, Hutchins AM (2012). Bean and rice meals reduce postprandial glycemic response in adults with type 2 diabetes: a cross-over study. Nutr J.

[68] Gravel K, Lemieux S, Asselin G, Dufresne A, Lemay A, Forest J-C, Dodin S (2010). Effects of pulse consumption in women presenting components of the metabolic syndrome: a randomized controlled trial. Med J Nutrition Metab.

[69] Kim Y, Keogh JB, Clifton PM (2017). Effects of two different dietary patterns on inflammatory markers, advanced glycation end products and lipids in subjects without type 2 diabetes: a randomised crossover study. Nutrients.

[70] Marinangeli CPF, Jones PJH (2011). Whole and fractionated yellow pea flours reduce fasting insulin and insulin resistance in hypercholesterolaemic and overweight human subjects. Br J Nutr.

[71] Pittaway JK, Ahuja KD, Robertson IK, Ball MJ (2007). Effects of a controlled diet supplemented with chickpeas on serum lipids, glucose tolerance, satiety and bowel function. J Am Coll Nutr.

[72] Saraf-Bank S, Esmaillzadeh A, Faghihimani E, Azadbakht L (2016). Effects of legume-enriched diet on cardiometabolic risk factors among individuals at risk for diabetes: a crossover study. J Am Coll Nutr.

[73] Tonstad S, Malik N, Haddad E (2014). A high-fibre bean-rich diet versus a low-carbohydrate diet for obesity. J Hum Nutr Diet.

[74] Tovar J, Nilsson A, Johansson M, Björck I (2014). Combining functional features of whole-grain barley and legumes for dietary reduction of cardiometabolic risk: a randomised cross-over intervention in mature women. Br J Nutr.

[75] Venn BJ, Perry T, Green TJ, Skeaff CM, Aitken W, Moore NJ, Mann JI, Wallace AJ, Monro J, Bradshaw A (2010). The effect of increasing consumption of pulses and wholegrains in obese people: a randomized controlled trial. J Am Coll Nutr.

[76] Winham DM, Hutchins AM, Johnston CS (2007). Pinto bean consumption reduces biomarkers for heart disease risk. J Am Coll Nutr.

[77] Hassanzadeh-Rostami Z, Hemmatdar Z, Pishdad GR, Faghih S (2019). Moderate consumption of red meat, compared to soy or non-soy legume, has no adverse effect on cardio-metabolic factors in patients with type 2 diabetes. Exp Clin Endocrinol Diabetes.

[78] Hosseinpour-Niazi S, Mirmiran P, Fallah-Ghohroudi A, Azizi F (2015). Non-soya legume-based therapeutic lifestyle change diet reduces inflammatory status in diabetic patients: a randomised cross-over clinical trial. Br J Nutr.

[79] Islam MM, Kamruzzaman M, Islam MS, Elahi MT, Rahman SS, Paul DK, Chaudhury MAZ, Rouf SA, Samad MA (2015). Impact of bread made from mix cereals and pulses on the glycemic profile in type 2 diabetic patients - A randomized controlled trial. Curr Nutr food Sci.

[80] Jang Y, Lee JH, Kim OY, Park HY, Lee SY (2001). Consumption of whole grain and legume powder reduces insulin demand, lipid peroxidation, and plasma homocysteine concentrations in patients with coronary artery disease: randomized controlled clinical trial. Arterioscler Thromb Vasc Biol.

[81] Jenkins DJ, Kendall CW, Augustin LS, Mitchell S, Sahye-Pudaruth S, Blanco Mejia S, Chiavaroli L, Mirrahimi A, Ireland C, Bashyam B (2012). Effect of legumes as part of a low glycemic index diet on glycemic control and cardiovascular risk factors in type 2 diabetes mellitus: a randomized controlled trial. Arch Intern Med.

[82] Jimenez-Cruz A, Bacardi-Gascon M, Turnbull WH, Rosales-Garay P, Severino-Lugo I (2003). A flexible, low-glycemic index mexican-style diet in overweight and obese subjects with type 2 diabetes improves metabolic parameters during a 6-week treatment period. Diabetes Care.

[83] Jiménez-Cruz A, Turnbull WH, Bacardi-Gascón M, Rosales-Garay P (2004). A high-fiber, moderate-glycemic-index, Mexican style diet improves dyslipidemia in individuals with type 2 diabetes. Nutr Res.

[84] Kang R, Kim M, Chae JS, Lee SH, Lee JH (2014). Consumption of whole grains and legumes modulates the genetic effect of the APOA5 -1131C variant on changes in triglyceride and apolipoprotein A-V concentrations in patients with impaired fasting glucose or newly diagnosed type 2 diabetes. Trials.

[85] Kim M, Song G, Kang M, Yoo HJ, Jeong TS, Lee SH, Lee JH (2016). Replacing carbohydrate with protein and fat in prediabetes or type-2 diabetes: greater effect on metabolites in PBMC than plasma. Nutr Metab.

[86] Liu Y, Wang Q, Li S, Yue Y, Ma Y, Ren G (2018). Convenient food made of extruded adzuki bean attenuates inflammation and improves glycemic control in patients with type 2 diabetes: a randomized controlled trial. Ther Clin Risk Manag.

[87] Winham DM, Hutchins AM (2007). Baked bean consumption reduces serum cholesterol in hypercholesterolemic adults. Nutr Res.

[88] Berry SE, Valdes AM, Drew DA, Asnicar F, Mazidi M, Wolf J, Capdevila J, Hadjigeorgiou G, Davies R, Al Khatib H (2020). Human postprandial responses to food and potential for precision nutrition. Nat Med.

[89] Hollander PA, Kushner PJPm, (2010). Type 2 diabetes comorbidities and treatment challenges: rationale for DPP-4 inhibitors. Postgrad Med.

[90] Riddle M, Umpierrez G, DiGenio A, Zhou R, Rosenstock JJDC (2011). Contributions of basal and postprandial hyperglycemia over a wide range of A1C levels before and after treatment intensification in type 2 diabetes. Diabetes Care.

[91] Sherifali D, Nerenberg K, Pullenayegum E, Cheng JE, Gerstein HCJDc, (2010). The effect of oral antidiabetic agents on A1C levels: a systematic review and meta-analysis. Diabetes Care.

[92] Derr R, Garrett E, Stacy GA, Saudek CDJDc, (2003). Is HbA1c affected by glycemic instability?. Diabetes Care.

[93] Edwards CH, Ryden P, Mandalari G, Butterworth PJ, Ellis PR (2021). Structure–function studies of chickpea and durum wheat uncover mechanisms by which cell wall properties influence starch bioaccessibility. Nature Food.

[94] Aldwairji MA, Chu J, Burley VJ, Orfila C (2014). Analysis of dietary fibre of boiled and canned legumes commonly consumed in the United Kingdom. J Food Compost Anal.

[95] Edwards CH, Warren FJ, Campbell GM, Gaisford S, Royall PG, Butterworth PJ, Ellis P (2015). A study of starch gelatinisation behaviour in hydrothermally-processed plant food tissues and implications for in vitro digestibility. Food Funct.

[96] Prasad V, Adapa D, Vana DR, Choudhury A, Asadutullah J, Chatterjee A (2018). Nutritional components relevant to type-2-diabetes: dietary sources metabolic functions and glycaemic effects. JRMDS.

[97] Mandaliya D, Patel S, Seshadri S, Rani VYU (2018). Fiber in our diet and its role in health and disease. Functional food and human health.

[98] Meyer KA, Kushi LH, Jacobs DR, Slavin J, Sellers TA, Folsom AR (2000). Carbohydrates, dietary fiber, and incident type 2 diabetes in older women. Am J Clin Nutr.

[99] Weickert MO, Pfeiffer AFH (2018). Impact of dietary fiber consumption on insulin resistance and the prevention of type 2 diabetes. J Nutr.

[100] Rizkalla SW, Bellisle F, Slama G (2002). Health benefits of low glycaemic index foods, such as pulses, in diabetic patients and healthy individuals. Br J Nutr.

[101] American Diabetes Association (2001). Postprandial blood glucose. Diabetes Care.

[102] Viguiliouk E, Glenn AJ, Nishi SK, Chiavaroli L, Seider M, Khan T, Bonaccio M, Iacoviello L, Mejia SB, Jenkins DJ (2019). Associations between dietary pulses alone or with other legumes and cardiometabolic disease outcomes: an umbrella review and updated systematic review and meta-analysis of prospective cohort studies. Adv Nutr.

