# Supplementary material for: Pulse consumption improves indices of glycemic control in adults with and without type 2 diabetes: a systematic review and meta-analysis of acute and long-term randomized controlled trials
Source: Eur J Nutr. 2021 Sep 29;61(2):809–24. doi: 10.1007/s00394-021-02685-y (PMC8854292; doi:10.1007/s00394-021-02685-y)
Supplement: Supplementary file 1 — Supplementary file1 (DOC 335 KB) [file 394_2021_2685_MOESM1_ESM.doc]

**Supplemental document**

Pulse consumption improves indices of glycemic control in adults with and without type 2 diabetes: a systematic review and meta-analysis of acute and long-term randomized controlled trials

Maryam S. Hafiz^1,2^, Matthew D. Campbell^3,4,5^, Lauren L. O’Mahoney^3^, Melvin Holmes^1^, Caroline Orfila^1^, Christine Boesch^1^*

^1^School of Food Science and Nutrition, University of Leeds, Leeds, United Kingdom; ^2^Faculty of Applied Medical Sciences, Department of Clinical Nutrition, King Abdul-Aziz University, Jeddah, Saudi Arabia; ^3^Faculty of Health Sciences and Wellbeing, University of Sunderland; ^4^Wellcome-MRC Institute of Metabolic Science, University of Cambridge; ^5^Leeds Institute of Cardiovascular and Metabolic Medicine, University of Leeds; ^6^Leicester Diabetes Centre, University of Leicester.

*Corresponding author: C Boesch, School of Food Science and Nutrition, University of Leeds, Leeds, LS2 9JT, United Kingdom. Tel +44 113 3430268. Email: [c.bosch@leeds.ac.uk](mailto:c.bosch@leeds.ac.uk)

Supplemental Table 1. Predetermined search terms

| **Search category** | **Search terms used** |
| --- | --- |
| 1. **Population** | (normoglycaemic) OR (normoglycaemia) OR (normoglycemia) OR (healthy) OR (normal) OR (diabetic) OR (diabetes) OR (T2DM) OR (T2D) OR (NIDDM) OR (adults) |
| 1. **Intervention** | (pulses) OR (pulse) OR (legumes) OR (leguminous) OR (legume) OR (beans) OR (bean) OR (peas) OR (pea) OR (chickpeas) OR (chickpea) OR (lentils) OR (lentil) OR (gram) |
| 1. **Outcomes** | (glucose) OR (FPG) OR (PPG) OR (glycemic) OR (glycaemia) OR (glycemia) OR (insulin) OR (post-prandial) OR (postprandial) OR (PPGR) OR (OGTT) OR (insulinemic) OR (insulinaemic) OR (HOMA) OR (Homeostatic Model Assessment of Insulin Resistance ) OR insulin resistance) OR (IR) OR (glycated hemoglobin) OR (HbA1c) OR (A1C) OR (A1c) OR (glycated haemoglobin) OR (glycosylated haemoglobin) |
| 1. **Combined search** | 1. AND (2) AND (3) |

Supplemental Table 2. Risk of bias assessment

| **Study** | **Risk of bias assessment** | | | | | |
| --- | --- | --- | --- | --- | --- | --- |
|  | **Domain** | | | | | Overall |
|  | *Randomization process* | *Deviation from intended intervention* | *Missing outcome data* | *Measurement of the outcome* | *Selection of the reported results* |  |
| **abete, 2008** | some concerns | low risk | low risk | low risk | some concerns | some concerns |
| **Abete, 2009** | some concerns | low risk | low risk | low risk | some concerns | some concerns |
| **Abeysekara, 2012** | low risk | low risk | some concerns | low risk | low risk | some concerns |
| **Agustia, 2019** | some concerns | low risk | some concerns | low risk | some concerns | some concerns |
| **Akhtar, 2019** | some concerns | low risk | some concerns | low risk | some concerns | some concerns |
| **Alizadeh, 2014** | some concerns | low risk | low risk | low risk | some concerns | some concerns |
| **Anderson, 1984** | some concerns | low risk | some concerns | low risk | some concerns | high risk |
| **Anderson, 2014** | some concerns | low risk | low risk | low risk | some concerns | some concerns |
| **Anguah, 2014** | some concerns | low risk | low risk | low risk | low risk | some concerns |
| **Augustin, 2016** | some concerns | low risk | low risk | low risk | some concerns | some concerns |
| **Boers, 2017** | low risk | low risk | low risk | low risk | low risk | low risk |
| **Bornet, 1987** | low risk | low risk | low risk | low risk | some concerns | some concerns |
| **Bornet, 1989** | some concerns | low risk | some concerns | low risk | some concerns | high risk |
| **Cryne, 2012** | some concerns | low risk | low risk | low risk | some concerns | some concerns |
| **Dandachy, 2018** | some concerns | low risk | low risk | low risk | some concerns | some concerns |
| **Dilwari, 1981** | some concerns | low risk | some concerns | low risk | some concerns | high risk |
| **Gravel, 2010** | low risk | low risk | low risk | low risk | some concerns | some concerns |
| **Greffeuil, 2015** | some concerns | low risk | low risk | low risk | some concerns | some concerns |
| **Hassanzadeh-Rostami, 2019** | low risk | low risk | low risk | low risk | some concerns | some concerns |
| **Hosseinpour-Niazi, 2015** | low risk | low risk | low risk | low risk | some concerns | some concerns |
| **Islam, 2015** | some concerns | low risk | low risk | low risk | some concerns | some concerns |
| **Jang, 2001** | some concerns | low risk | low risk | low risk | some concerns | some concerns |
| **Jenkins, 1980** | some concerns | low risk | some concerns | low risk | some concerns | high risk |
| **Jenkins, 1980** | some concerns | low risk | some concerns | low risk | some concerns | high risk |
| **Jenkins, 1982** | some concerns | low risk | low risk | low risk | some concerns | high risk |
| **Jenkins, 2012** | low risk | low risk | low risk | low risk | some concerns | some concerns |
| **Jimenez-Cruz, 2003** | some concerns | low risk | low risk | low risk | some concerns | some concerns |
| **Jimenez-Cruz, 2004** | some concerns | low risk | low risk | low risk | some concerns | some concerns |
| **Johnson, 2005** | some concerns | low risk | low risk | low risk | some concerns | some concerns |
| **Kang, 2014** | some concerns | low risk | low risk | low risk | some concerns | some concerns |
| **Kim, 2014** | low risk | low risk | low risk | low risk | some concerns | some concerns |
| **Kim, 2016** | low risk | low risk | low risk | low risk | some concerns | some concerns |
| **Kim, 2017** | some concerns | low risk | low risk | low risk | some concerns | some concerns |
| **Liu, 2018** | low risk | low risk | low risk | low risk | some concerns | some concerns |
| **Mani, 1992** | some concerns | low risk | some concerns | low risk | some concerns | high risk |
| **Marinangeli, 2009** | some concerns | low risk | low risk | low risk | some concerns | some concerns |
| **Marinangeli, 2011** | some concerns | low risk | low risk | low risk | some concerns | some concerns |
| **Mehio, 1997** | some concerns | low risk | low risk | low risk | some concerns | some concerns |
| **Mollard, 2011** | low risk | low risk | low risk | low risk | some concerns | some concerns |
| **Moravek, 2018** | some concerns | low risk | low risk | low risk | some concerns | some concerns |
| **Nestel, 2004** | some concerns | low risk | low risk | low risk | some concerns | some concerns |
| **Olmedilla-Alonso, 2013** | some concerns | low risk | low risk | low risk | some concerns | some concerns |
| **Onyechi, 1998** | some concerns | low risk | some concerns | low risk | some concerns | high risk |
| **Pittaway, 2007** | some concerns | low risk | low risk | low risk | some concerns | some concerns |
| **Potter, 1981** | low risk | low risk | some concerns | low risk | low risk | some concerns |
| **Ramdath, 2017** | some concerns | low risk | low risk | low risk | some concerns | some concerns |
| **Ramdath, 2018** | some concerns | low risk | low risk | low risk | some concerns | some concerns |
| **Reverri, 2015** | some concerns | low risk | low risk | low risk | some concerns | some concerns |
| **Saraf-Bank, 2016** | some concerns | low risk | low risk | low risk | some concerns | some concerns |
| **Schafer, 2003** | some concerns | low risk | low risk | low risk | some concerns | some concerns |
| **Tappy, 1986** | some concerns | low risk | some concerns | low risk | some concerns | high risk |
| **Thompson, 2012** | low risk | low risk | low risk | low risk | some concerns | some concerns |
| **Tonstad, 2014** | some concerns | low risk | low risk | low risk | some concerns | some concerns |
| **Torsdottir, 1989** | some concerns | low risk | some concerns | low risk | some concerns | high risk |
| **Tovar, 2014** | low risk | low risk | low risk | low risk | some concerns | some concerns |
| **Traianedes, 1986** | some concerns | low risk | some concerns | low risk | some concerns | high risk |
| **Venn, 2010** | some concerns | low risk | some concerns | low risk | some concerns | high risk |
| **Winham, 2007** | low risk | low risk | low risk | low risk | some concerns | some concerns |
| **Winham, 2007** | low risk | low risk | low risk | low risk | some concerns | some concerns |
| **Winham, 2017** | some concerns | low risk | low risk | low risk | some concerns | some concerns |
| **Wong, 2009** | low risk | low risk | low risk | low risk | some concerns | some concerns |
| **Yoshimoto, 2020** | low risk | low risk | low risk | low risk | some concerns | some concerns |
| **Zafar, 2015** | some concerns | low risk | low risk | low risk | some concerns | some concerns |
| **Zhu, 2019** | low risk | low risk | low risk | low risk | some concerns | some concerns |
| **Zurbau, 2019** | low risk | low risk | low risk | low risk | some concerns | some concerns |

Supplemental Table 3. GRADE assessment

1. Due to high unexplained heterogeneity
2. Due to differences in control
3. Due to substantial differences in interventions and comparisons
4. The 95% CI included benefits as well as no effect
5. The 95% CI included benefits and harms

| **Outcome** | No. of trials | No. of participants | Certainty assessment | | | | | Effect estimate | Grade |
| --- | --- | --- | --- | --- | --- | --- | --- | --- | --- |
|  |  |  | Risk of bias | Inconsistency | Indirectness | Imprecision | Other considerations |  |  |
| **PPGR in normoglycemia** | 27 | 690 | Not serious | Serious ^a^ | Serious ^b^ | Not serious | none | -1.23 [-1.59, -0.88] | Low |
| **PPGR in T2DM** | 6 | 136 | Not serious | Serious ^a^ | Serious ^b^ | Not serious | none | -2.89 [-4.61, -1.18] | Low |
| **Fasting glucose healthy** | 16 | 1037 | Not serious | Not serious | Very serious ^c^ | Serious ^d^ | none | -0.06 [-0.12, 0.00] | Very low |
| **Fasting glucose DM** | 11 | 808 | Not serious | Serious ^a^ | Very serious ^c^ | Not serious | none | -0.51 [-0.79, -0.24] | Very low |
| **Hba1c DM** | 7 | 524 | Not serious | Serious ^a^ | Very serious ^c^ | Very serious ^e^ | None | -0.20 [-0.36, -0.05] | Very low |
| **HOMA** | 5 | 431 | Not serious | Serious ^a^ | Very serious ^c^ | Not serious | none | -0.47 [-0.80, -0.14] | Very low |


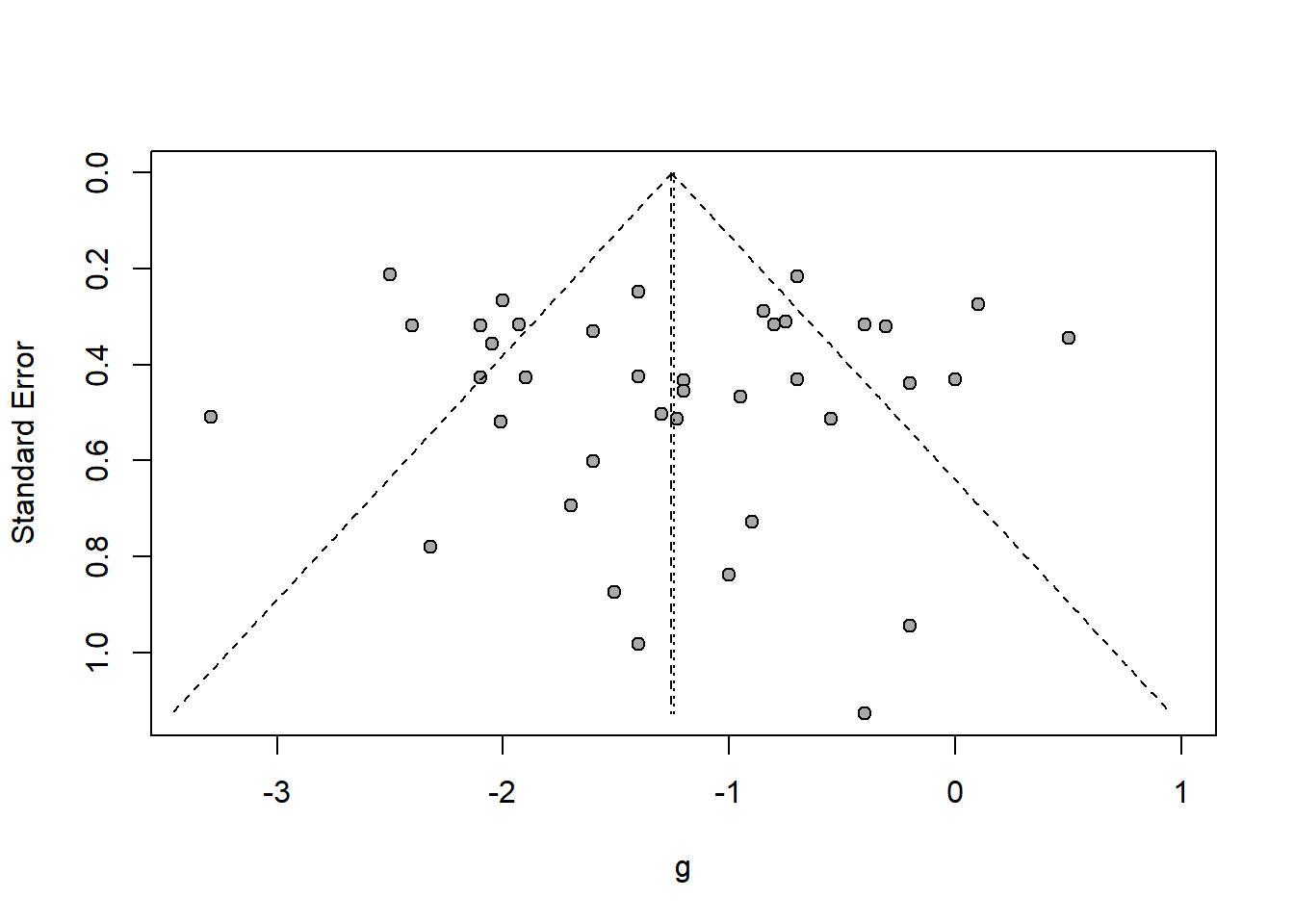


Supplemental Figure 1. funnel plot of acute studies included in meta-analysis investigating acute glucose response after pulse consumption in adults without T2DM


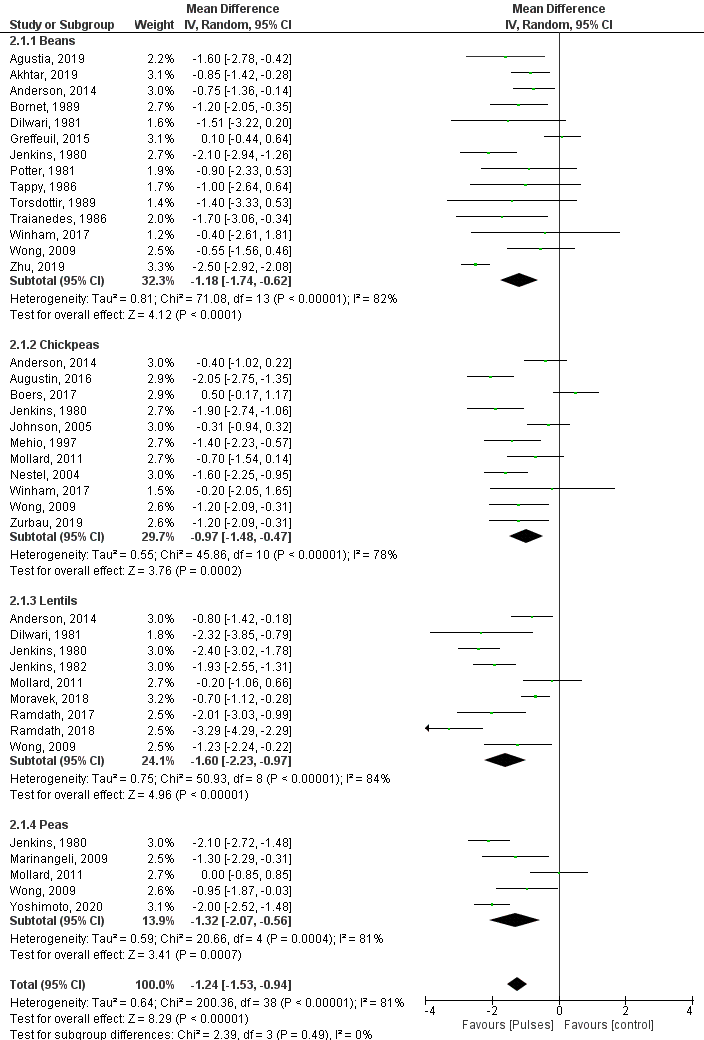


Supplemental Figure 2. Subgroup analysis by pulse type on acute studies investigating acute glucose response after pulse consumption in adults without T2DM


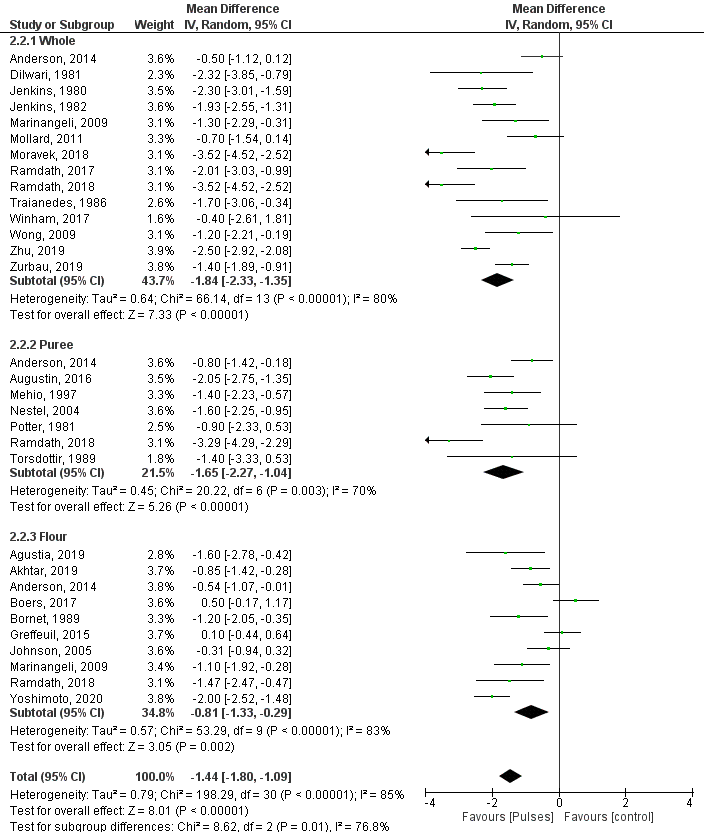


Supplemental Figure 3. Subgroup analysis by physical form of pulse on acute studies investigating acute glucose response after pulse consumption in adults without T2DM


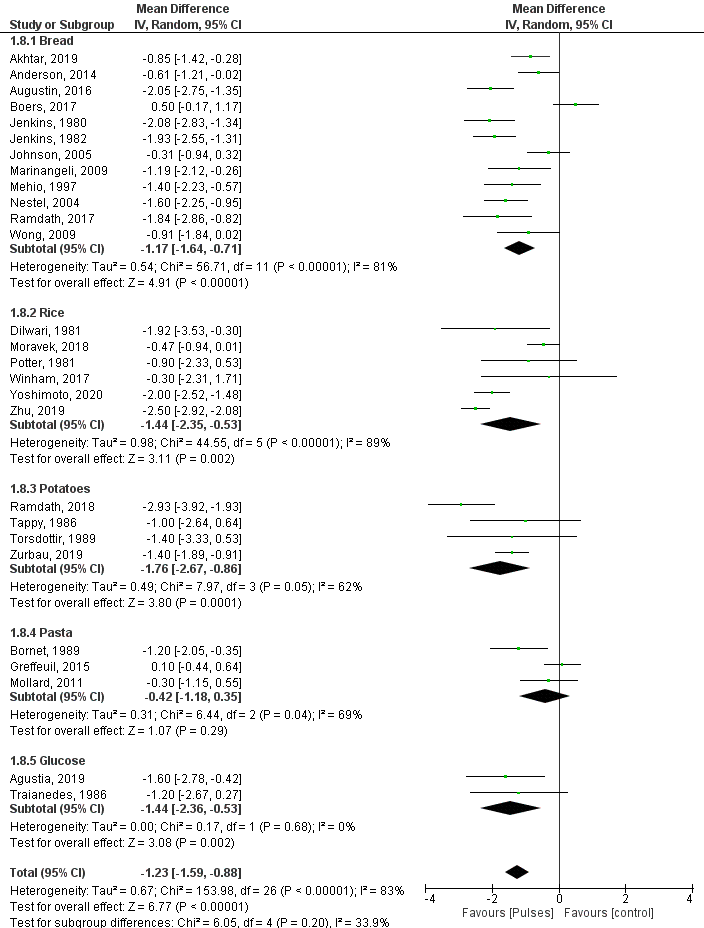


Supplemental Figure 4. Subgroup analysis by control group on acute studies investigating acute glucose response after pulse consumption in adults without T2DM


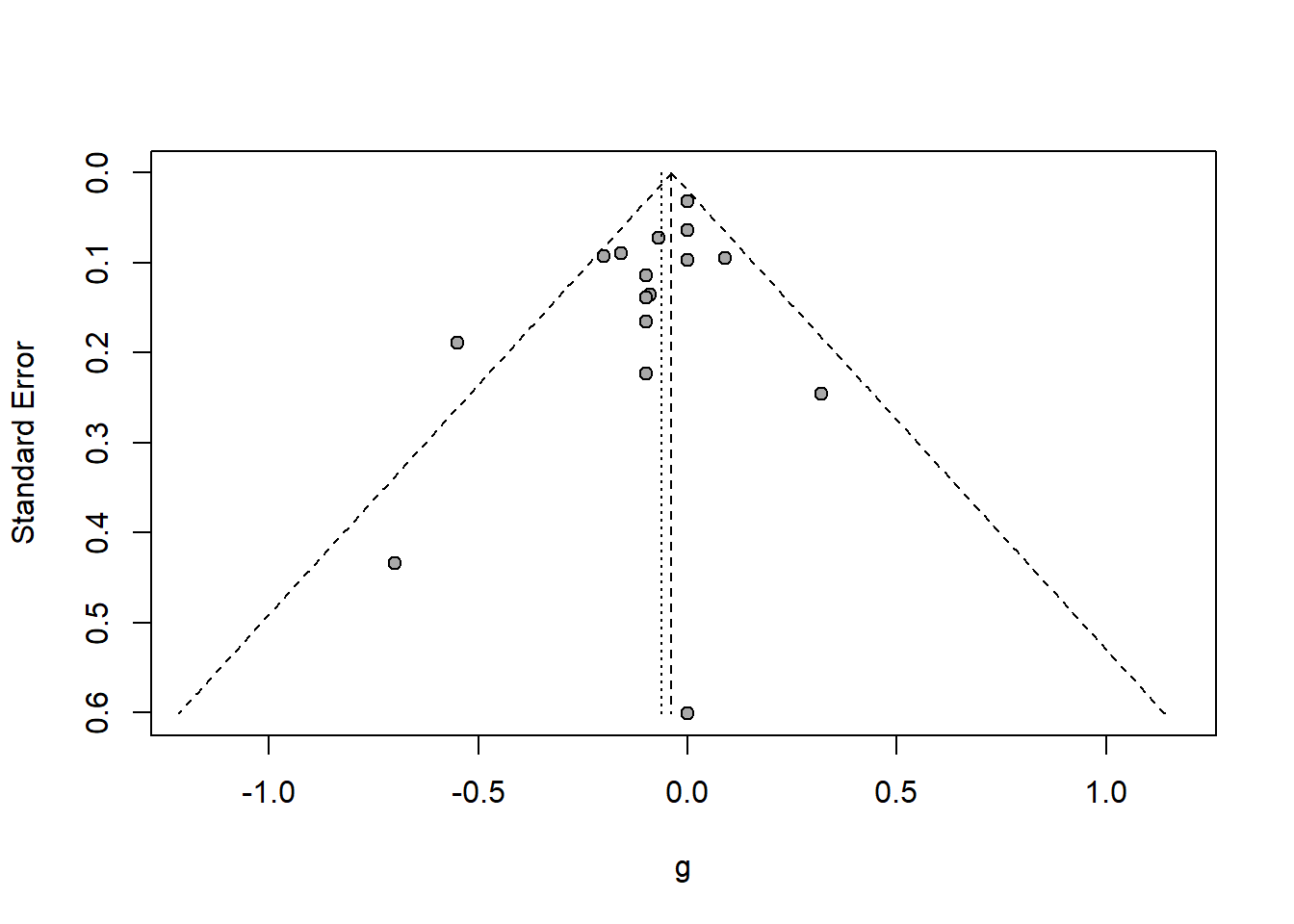


Supplemental Figure 6. Funnel plot of long-term trials included in meta-analysis investigating affect of pulse consumption in adults with T2DM

Supplemental Figure 5. Funnel plot of long-term trials included in meta-analysis investigating affect of pulse consumption in adults without T2DM


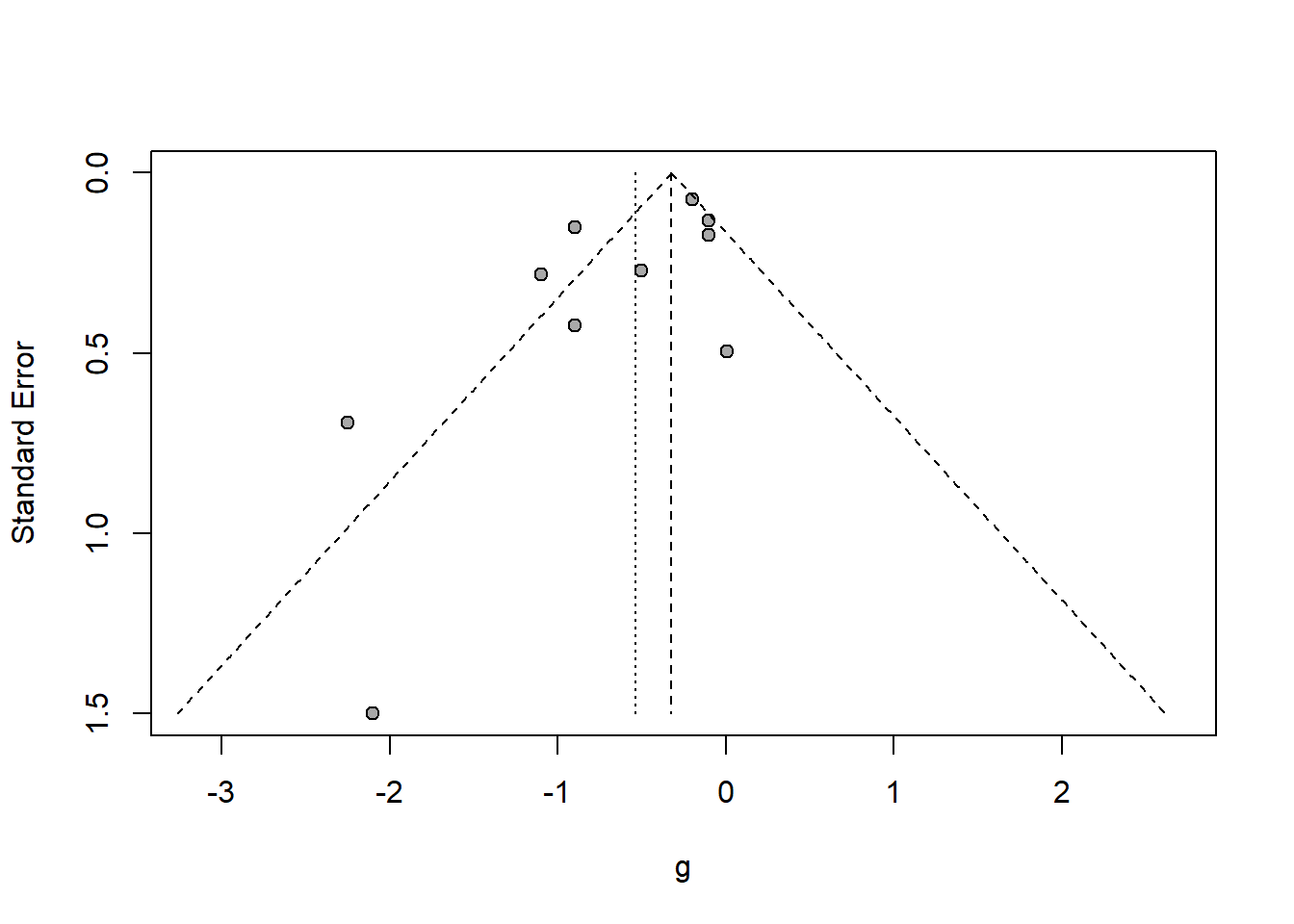


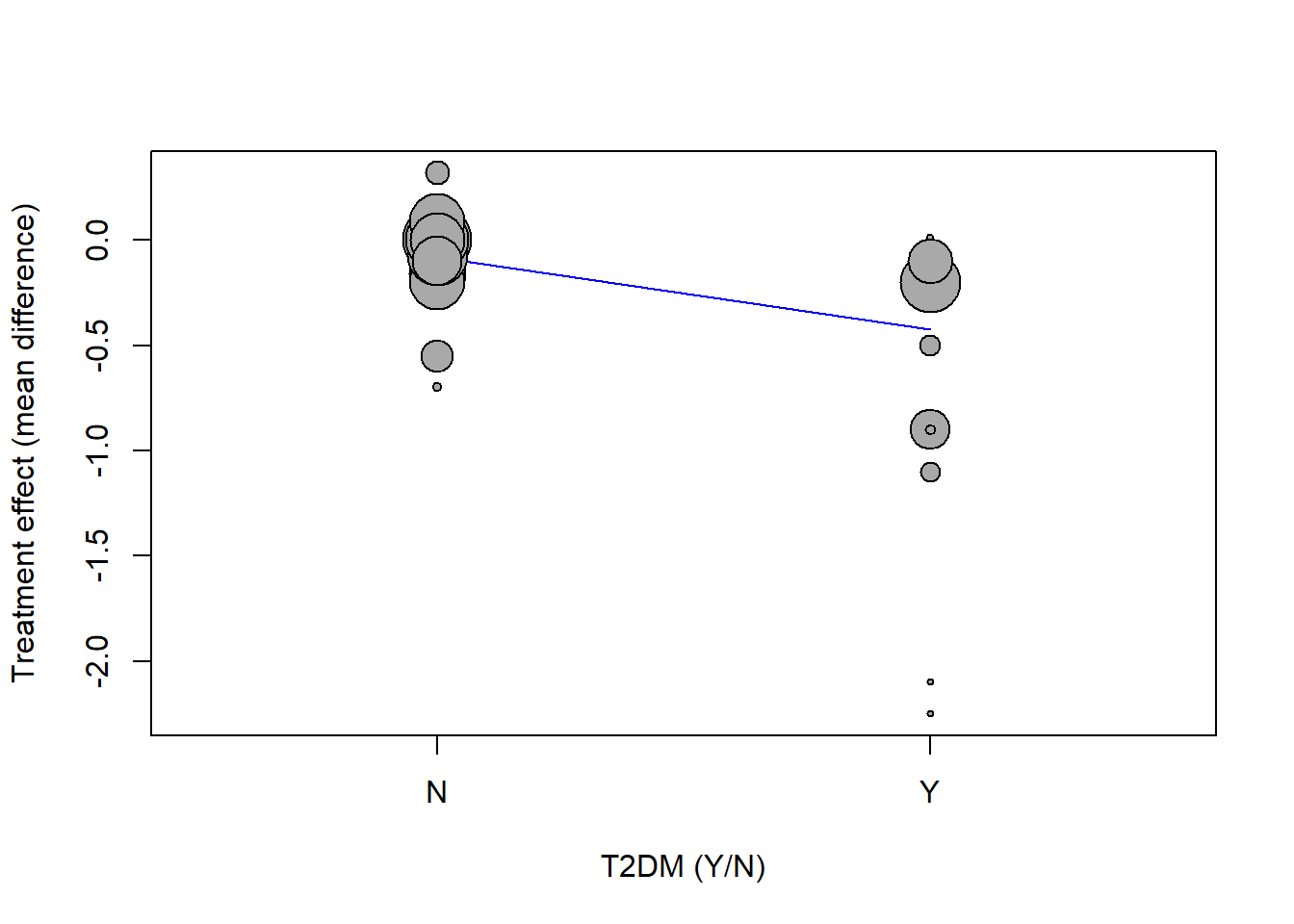


Supplemental Figure 7. Comparison of effect size in long-term trials between adults with and without T2DM


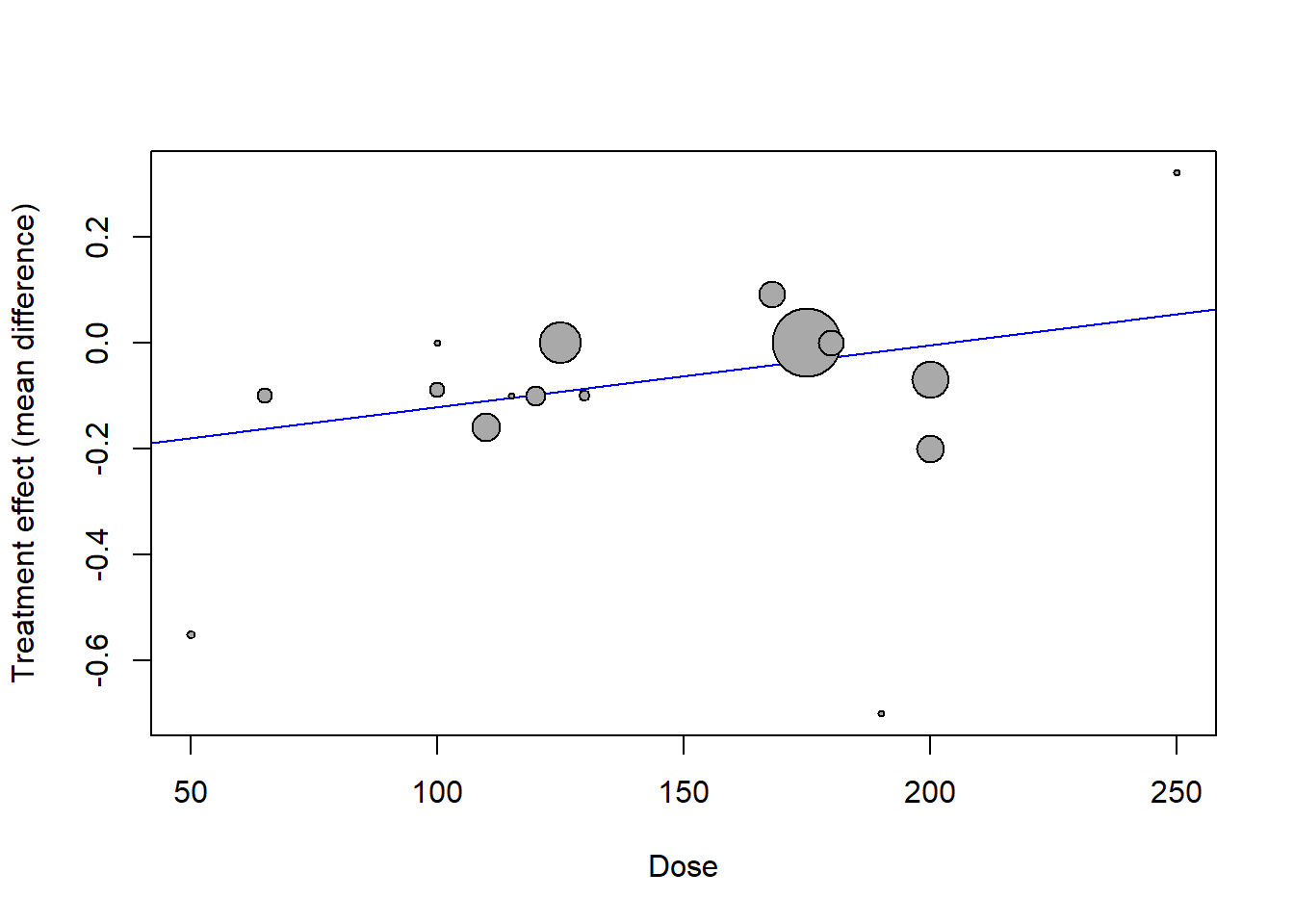


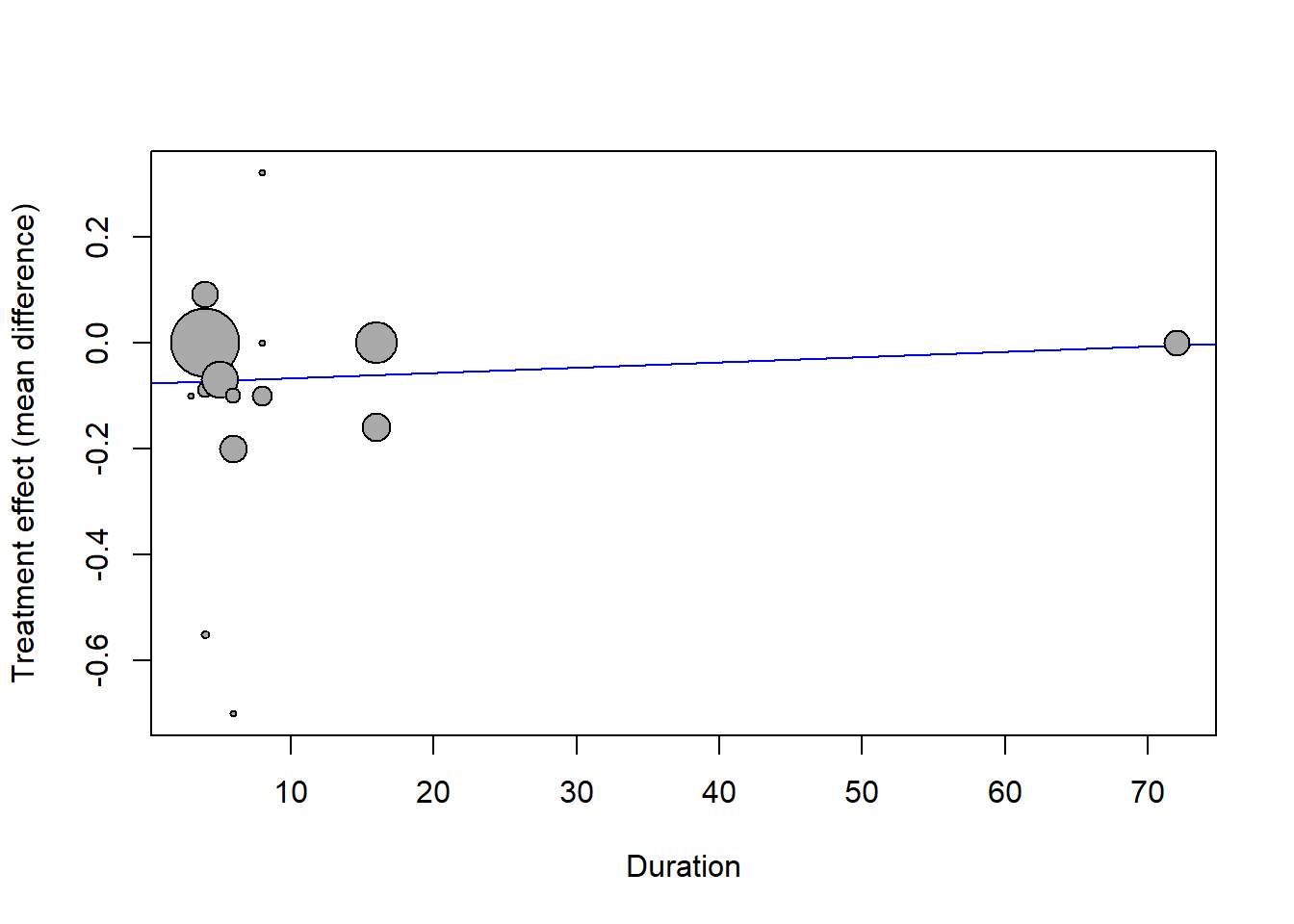


Supplemental Figure 8. Meta-regression of affect of dose and duration on modifying effect size in long-term trials on normoglycaemic adults


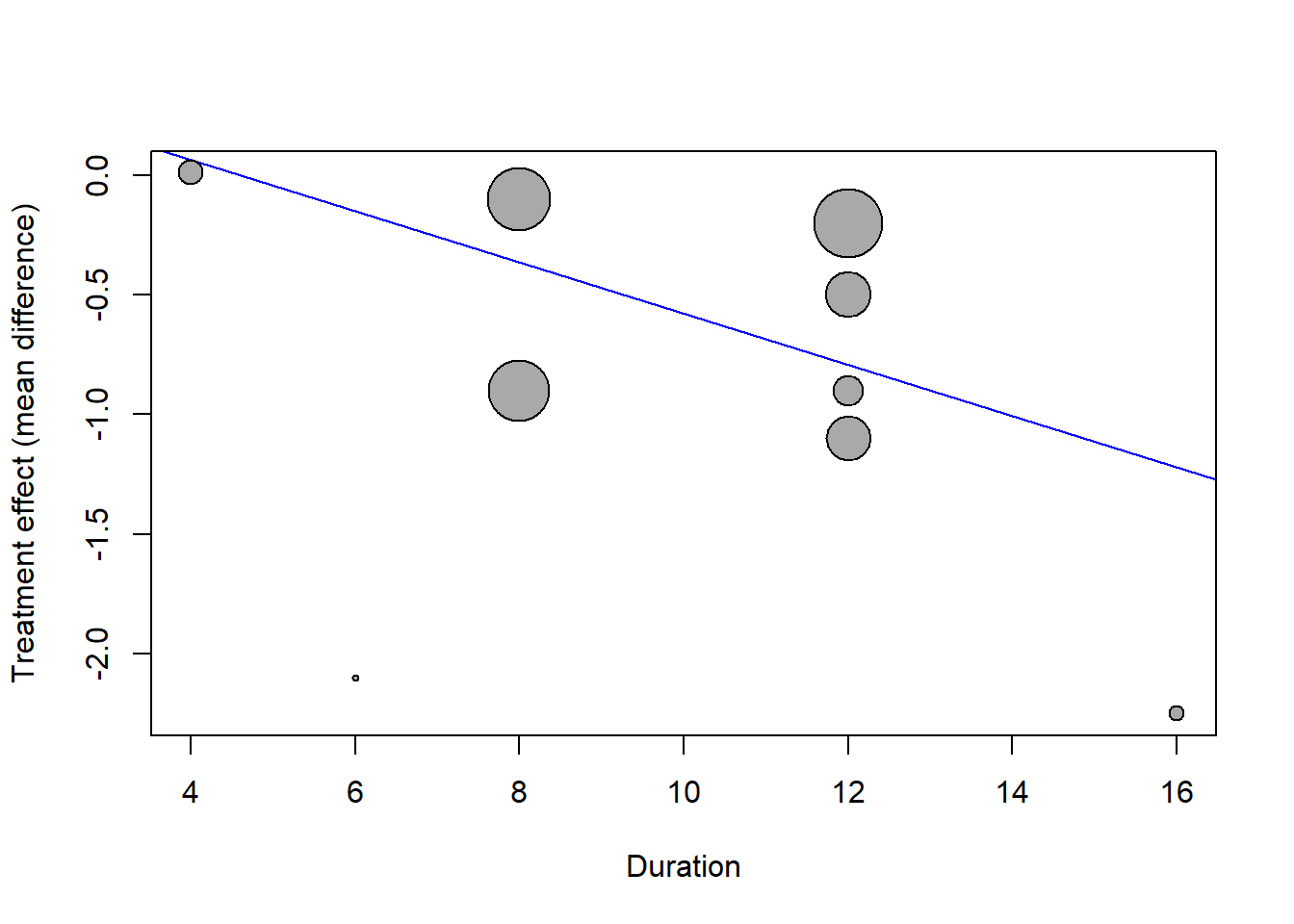

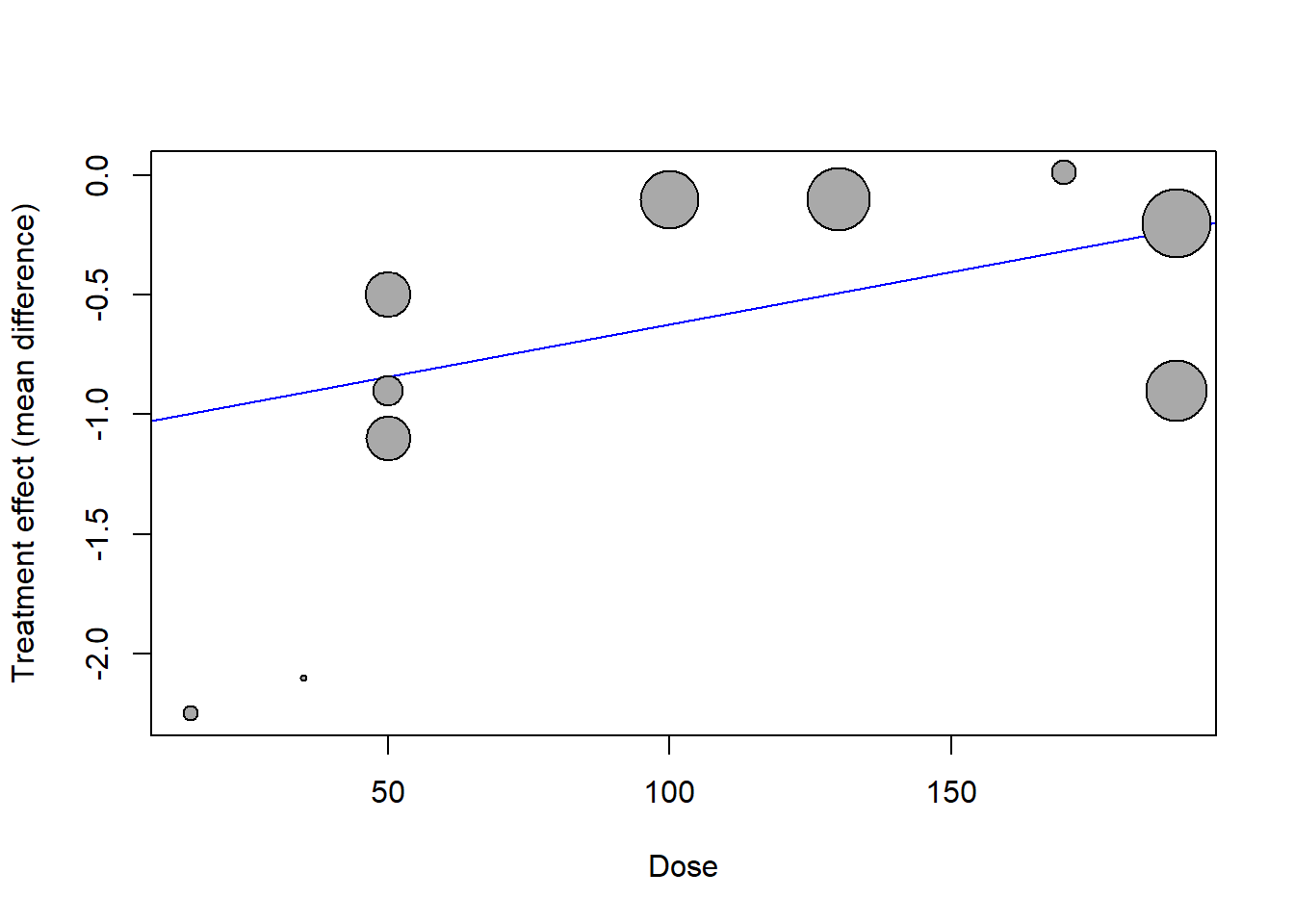


Supplemental Figure 9. Meta-regression of affect of dose and duration on modifying effect size in long-term trials on T2DM adults
